# Identification of Human Tissue Kallikrein 6 as a Potential Marker of Laryngeal Cancer Based on the Relevant Secretory/Releasing Protein Database

**DOI:** 10.1155/2014/594093

**Published:** 2014-02-11

**Authors:** Ying Zhang, Zaixing Zhang, Lei Yang, Bin Xu, Weihua Li, Pingzhang Tang, Zongmin Zhang, Naijun Han, Yanning Gao, Shujun Cheng, Ting Xiao

**Affiliations:** ^1^Department of Biochemistry and Molecular Biology, Molecular Medicine and Cancer Research Center, Chongqing Medical University, Chongqing 400016, China; ^2^State Key Laboratory of Molecular Oncology, Beijing Key Laboratory for Carcinogenesis and Cancer Prevention, Cancer Institute (Hospital), Peking Union Medical College & Chinese Academy of Medical Sciences, P.O. Box 2258, Beijing 100021, China; ^3^Department of Head and Neck Surgical Oncology, Cancer Institute (Hospital), Peking Union Medical College & Chinese Academy of Medical Sciences, Beijing 100021, China; ^4^National Center of Biomedical Analysis, Beijing 100021, China

## Abstract

*Objective*. This study was aimed to create a large-scale laryngeal cancer relevant secretory/releasing protein database and further discover candidate biomarkers. *Methods*. Primary tissue cultures were established using tumor tissues and matched normal mucosal tissues collected from four laryngeal cancer patients. Serum-free conditioned medium (CM) samples were collected. These samples were then sequentially processed by SDS-PAGE separation, trypsin digestion, and LC-MS/MS analysis. The candidates in the database were validated by ELISA using plasma samples from laryngeal cancer patients, benign patients, and healthy individuals. *Results*. Combining MS data from the tumor tissues and normal tissues, 982 proteins were identified in total; extracellular proteins and cell surface proteins accounted for 15.0% and 4.3%, respectively. According to stringent criteria, 49 proteins were selected as candidates worthy of further validation. Of these, human tissue kallikrein 6 (KLK6) was verified. The level of KLK6 was significantly increased in the plasma samples from the cancer cohort compared to the benign and healthy cohorts and moreover showed a slight decrease in the postoperative plasma samples in comparison to the preoperative plasma samples. *Conclusions*. This laryngeal cancer-derived protein database provides a promising repository of candidate blood biomarkers for laryngeal cancer. The diagnostic potential of KLK6 deserves further investigation.

## 1. Introduction

An estimated 640 000 new cases and 360 000 deaths from head and neck cancer occurred in 2011 worldwide [1]. Head and neck cancer is a type of epithelial malignancy that arises in the paranasal sinuses, nasal cavity, oral cavity, pharynx, and larynx [2], with laryngeal cancer representing approximately 20% of the cancer burden. The overwhelming majority of laryngeal cancer is squamous cell carcinomas, and the most important risk factors are tobacco use and alcohol consumption. A slight increase in the incidence of laryngeal cancer has been detected in Shanghai, from 1.29 per 100 000 in 1972 to 4.3 per 100 000 in 2002 [3]. In the past three decades, clinical treatment with functional organ preservation has substantially improved the quality of life but not overall survival of patients. The 5-year survival rate for all stages is 60–70% yet approaches 90% for the early stage (stages I and II). The discrepancy is largely attributed to a lack of suitable biomarkers for the early detection of primary and relapsed tumors. Therefore, early detection should significantly reduce the cancer burden. Unfortunately, approximately 40% of all patients present with advanced stages (stages III and IV), due to the fact that the disease arises in asymptomatic changes.

There are limitations in the early detection of laryngeal cancer, particularly, the supraglottic and subglottic histological types. In addition to expensive medical imaging techniques, tumor biomarkers assist in clinical decisionmaking. However, some serum tumor markers, such as carcinoembryonic antigen (CEA), squamous cell carcinoma antigen (SCC-Ag), soluble cytokeratin 19 fragments (CYFRA 21-1), and tissue polypeptide antigen (TPA), continue to be utilized in clinic despite their poor sensitivity and specificity. The lack of proven screening modalities for laryngeal cancer necessitates the search for diagnostic biomarkers for the early detection of this malignancy.

The proteomic analysis of relevant cancer samples is a powerful tool to identify laryngeal cancer biomarkers. In addition to tissues [4–6], body fluids are the most widely used specimens in biomarker discovery and include blood [7, 8] and saliva [9, 10]. Secretome analyses using laryngeal cancer cell lines have also helped to reveal candidate biomarkers [11, 12]. Nonetheless, biomarker discovery studies remain imperfect, primarily due to the high complexity of the body fluid-associated proteome and the reduced physiological relevance of the cell line associated secretome. A novel approach based on the proteomic analysis of serum-free conditioned media derived from primary tissue cultures was previously developed to resolve these issues. The model of primary tissue culture is more similar to the physiological status *in vivo*, and a serum-free conditioned medium-relevant proteome is less complex. To date, such as approach has been successfully utilised to screen candidate biomarkers for lung cancer and ovarian cancer [13, 14].

In the present study, primary tissue cultures from laryngeal cancer patients were produced and an MS-based proteomic analysis of their corresponding serum-free CM samples was performed to generate a laryngeal cancer-derived secretory/releasing proteome of 982 CM proteins. A list of candidate biomarkers was chosen according to a set of criteria, highlighting KLK6, a protein that belongs to the 15-member KLK family in which KLK3 (also known as prostate-specific antigen) is the most useful marker for the early detection of prostate cancer. The diagnostic potential of KLK6 was then validated in circulating plasma samples from laryngeal cancer patients, benign patients, and healthy individuals.

## 2. Materials and Methods

### 2.1. Patients and Specimens

All specimens were obtained from patients recruited between September 2008 and April 2011 at the Cancer Hospital, Peking Union Medical College (PUMC) and Chinese Academy of Medical Sciences (CAMS), with written informed consent and the approval of the Ethics Committee of Cancer Institute and Hospital (no. 12-130/664).

For primary tissue cultures, the tumor tissues and matched normal tissues were collected from four patients with primary laryngeal cancer who underwent surgical resection. All of the patients had never accepted any type of therapy prior to surgery.

The histological information concerning all the tissue samples, with the malignancies all being squamous cell carcinomas (SCC), was confirmed by pathologists. The corresponding clinicopathological data are shown in Table S1 in Supplementary Material available online at http://dx.doi.org/10.1155/2014/594093.

For ELISA, peripheral blood samples were obtained from 149 patients with laryngeal SCC (140 males and 9 females; median age of 58 years old), 145 patients with benign head and neck disease (42 males and 103 females; median age of 52 years old), and 124 healthy cases (102 males and 22 females; median age of 48 years old) from a routine “health screening” program at the Cancer Hospital, PUMC and CAMS. None of the patients had accepted surgical, radiotherapy, or chemotherapy, prior to blood sampling. The demographics and clinical characteristics of the cancer cohort are described in supplementary Table S2. Additionally, 70 cases of preoperative and postoperative blood samples were prepared; the postoperative blood samples were collected from the patients a week after surgery. The corresponding clinicopathological data are presented in Table S3. The circulating plasma samples were collected according to the methods described in a previous study [14].

### 2.2. Primary Tissue Culture and CM Preparation

Primary tissue cultures for tumor tissues and the matched histologically normal tissues from 4 laryngeal SCC patients were produced. The experimental procedures were well-described in a previous study [14]. In brief, fresh tissue samples were rinsed, cut into about 2 mm^3^ pieces, and then transferred to 60 mm culture dishes. The explants were placed on a rocking platform and cultivated at 37°C in LHC-9 medium (serum-free), in a gas mixture of 50% O_2_, 45% N_2_, and 5% CO_2_. 24 hours later, the explants were incubated in the CM, serum-free LHC-9 without bovine pituitary extract, for another 24 h. The CM was subsequently collected, dialyzed, and lyophilized prior to gel separation. In addition, explant samples from normal tissue and tumor tissue for each case were randomly chosen and fixed in formalin, embedded in paraffin, cut into slices, and stained with hematoxylin and eosin. The histology and integrity of the tissues were evaluated by pathologists.

### 2.3. SDS-PAGE Separation and In-Gel Trypsin Digestion of CM Proteins

Aliquots of 50 *μ*g total protein from the CM samples were separated by 10% SDS-PAGE. After staining with the Coomassie Blue, the gel was cut into distinct sample lanes, and each sample lane was sliced into 50 pieces, top to bottom from the loading well. In-gel trypsin digestion was then performed according to the protocol described by Shevchenko et al. [15].

### 2.4. LC-MS/MS Analysis of CM Proteins

The detailed procedure of LC-MS/MS analysis was described in a previous study [14]. Peptide extracts were dissolved in 6 *μ*L 0.1% formic acid and separated on a nanoACQUITY system (Waters, MA, USA) equipped with a Symmetry C18 5 *μ*m, 180 *μ*m × 20 mm precolumn and an ethylene bridged hybrid (BEH) C18 1.7 *μ*m, 75 *μ*m × 250 mm, analytical reversed-phase column (Waters, MA, USA). The samples were initially transferred with an aqueous 0.1% formic acid solution to the precolumn at a flow rate of 7 *μ*L/min for 3 min. Mobile phase A was water with 0.1% formic acid, and mobile phase B was 0.1% formic acid in acetonitrile. The peptides were separated with a linear gradient of 97–60% mobile phase A and 3–40% mobile phase B over 90 min at 200 nl/min followed by 10 min at 10% mobile phase A and 90% mobile phase B. The column was reequilibrated at initial conditions for 20 min. The column temperature was maintained at 35°C. The lock mass was delivered from the auxiliary pump of the nanoACQUITY pump with a constant flow rate of 300 nl/min at a concentration of 100 fmol/*μ*L Glu-fibrinopeptide B. Analysis of tryptic peptides was performed using a SYNAPT high definition mass spectrometer (Waters, MA, USA). For all measurements, the mass spectrometer was operated in the v-mode with a typical resolving power of at least 10,000 full-width half-maximum. The TOF analyzer of the mass spectrometer was calibrated with the MS/MS fragment ions of Glu-fibrinopeptide B from *m*/*z* 50 to 1600. The reference sprayer was sampled with a frequency of 30 s. Accurate mass LC-MS data were collected in high definition MSE mode (low collision energy, 4 eV; high collision energy, ramping from 15 to 45 eV; switching every 1 s; interscan time, 0.02 s). The mass range was from *m*/*z* 300 to 1990.

### 2.5. Data Searching and Bioinformatic Analysis

ProteinLynx GlobalServer version 2.3 (Waters, MA, USA) was used to create peak list files. Database searches were performed with the pFind 2.4 search engine (http://pfind.ict.ac.cn/) against Uniprot Knowledgebase Release 12.6 (human, 76137 entries). The search criteria for the peptide and protein identification were reported previously [14]. In brief, two missed cleavages were allowed, carbamidomethylation (Cys) was set as a fixed modification, oxidation (Met) was set as a variable modifications, precursor ion mass tolerance was 0.2 Da, fragments ion mass tolerance was set to 0.3 Da, and FDR was set as 1% by default. The substantial properties of the CM protein database were broadly analyzed with the BiNGO tool [16].

### 2.6. Measurement of Kallikrein 6 (KLK6) in Circulating Plasma by ELISA

The KLK6 protein level in plasma was measured by double-antibody sandwich ELISA. The assay was performed using the ELISA kit (40365, Yuanye Bio-Technology Co., Ltd, Shanghai, China) according to the manufacturer's instructions.

### 2.7. Statistical Analysis

All calculations were performed with SPSS software, version 17.0 (SPSS INC, IL, USA). Comparisons of the plasma KLK6 levels between two independent sample cohorts were assessed by the Mann-Whitney *U* test, and those among more than two independent cohorts were assessed by the Kruskal-Wallis *H* test. The comparison of the KLK6 levels between paired samples was evaluated by the Wilcoxon test. All comparisons were two-tailed, and *P* values less than 0.05 were considered to be statistically significant. The diagnostic performance of the plasma KLK6 levels was evaluated with a receiver operating characteristic (ROC) curve.

## 3. Results

### 3.1. Establishment of a Laryngeal SCC-Related CM Protein Database

Tissue cultures were established from tumor tissues and matched normal tissues of four laryngeal SCC patients and were based on a serum-free primary culture system. These tissue samples essentially maintained histological integrity during the two days of culturing, with only approximately 20% necrosis area, as shown in Figure S1. Their CM samples were prepared for further proteomic analysis. The protein identifications in the four paired CM samples were presented in Table S4. In total, 684 and 770 proteins were identified in the CM samples from the normal tissues and tumor tissues, respectively; with 472 overlapping proteins, they constituted a laryngeal cancer-derived secretory/releasing proteome with a total of 982 proteins.

**Figure 1:**
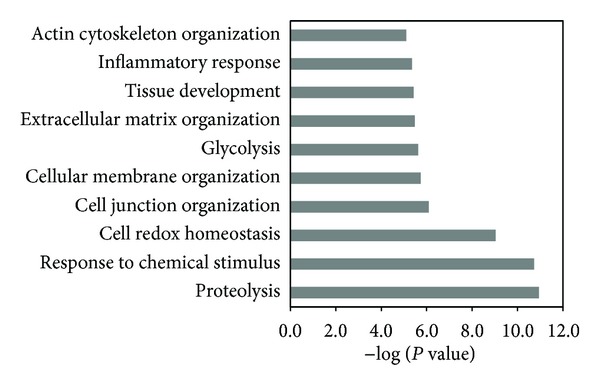
The major biological processes significantly enriched by the BiNGO tool. The top ten biological processes and their corresponding significance (negative of the *P* value), with the Benjamini & Hochberg False Discovery Rate correction for multiple testing and all human annotations as the reference set are shown on the *y*- and *x*-axes, respectively.

An exhaustive analysis of the characteristics of the total CM proteins in the secretory/releasing proteome was performed via GO enrichment. It was found that “proteins in extracellular region part” and “proteins in cell surface” were significantly enriched, accounting for 15.0% and 4.3% of the total CM proteins, respectively (Figure S2). Moreover, the total CM proteins mainly converged on these biological processes, for example, “proteolysis,” “cell redox homeostasis,” “cell junction organization,” “cellular membrane organization,” “glycolysis,” “extracellular matrix organization,” and “inflammatory response” (Figure 1).

### 3.2. Selection of Candidate Biomarkers

**Figure 2:**
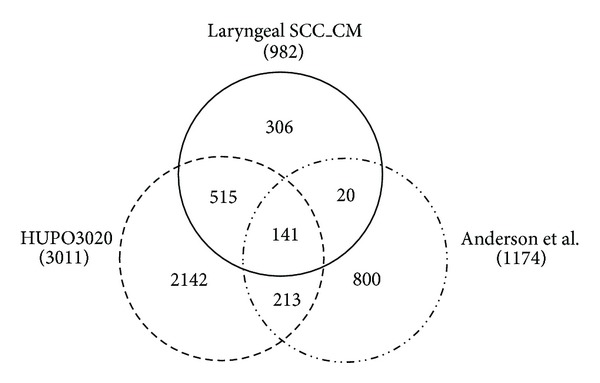
Comparisons of the laryngeal cancer-derived “secretory/releasing proteome” (laryngeal SCC_CM) with published human plasma proteomes. The number in parentheses indicates the number of proteins in the database.

Candidate biomarkers were selected from this CM protein database according to a series of stringent criteria. (1) The set of proteins existing in the reported human plasma proteomes was chosen. Comparing the list of the 982 CM proteins with the plasma proteomes published by HUPO3020 Plasma Proteome Project [17] and Anderson et al. [18], there was an overlap of 141 CM proteins in these two datasets (Figure 2). (2) Proteins found in more than one CM sample were prioritized. By applying this criterion, 30 CM proteins were removed, whereas 111 CM proteins remained for the next selection. (3) We further eliminated proteins that have been reported as serological markers of head and neck cancer. In accordance with the list of proteins previously studied in the serum of head and neck cancer patients (summarized in Table S5) [19–21], six CM proteins were removed, with 105 CM proteins remaining. (4) A set of extracellular and plasma membrane proteins was chosen, recognizing that these proteins are likely to enter the circulation in detectable levels; 40 CM proteins were removed, resulting in a shortened list of 65 CM proteins. (5) We further eliminated known high-abundance plasma proteins. Thus, the remaining 49 CM proteins represented candidate biomarkers of laryngeal SCC, as listed in Table S6. Given the availability of commercial ELISA kits, KLK6 was preferentially selected for further validation in plasma.

### 3.3. Analysis of KLK6 in the Plasma of Laryngeal SCC Patients and Control Cohorts

**Figure 3:**
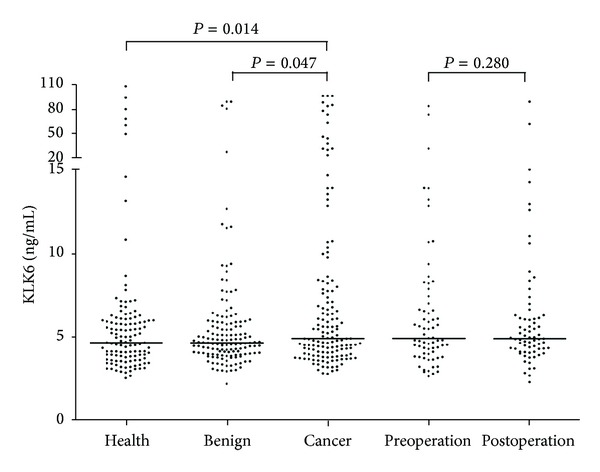
Scatter plots of the KLK6 protein levels in the five cohorts. Health: healthy individuals, benign: benign patients, cancer: cancer patients, preoperation: preoperative samples, and postoperation: postoperative samples. The bars denote the median levels of KLK6.

The levels of KLK6 were measured in circulating plasma samples from 124 healthy cases, 145 patients with benign head and neck disease, and 149 patients with laryngeal SCC. As shown in Table 1 and Figure 3, the plasma levels of KLK6 were significantly higher in the laryngeal SCC patients (median, interquartile range: 4.90, 4.26) than in the benign cohort (median, interquartile range: 4.65, 1.86; *P* = 0.047) and healthy cohort (median, interquartile range: 4.65, 2.32; *P* = 0.014), though no considerable difference was observed between the latter two cohorts (*P* = 0.607). With regard to the laryngeal SCC cohort, there was no significant correlation between the plasma KLK6 levels and clinicopathological characteristics, including anatomical region, disease stage, tumor size, lymph node metastasis, and tumor differentiation.

In addition, the levels of KLK6 were assessed in circulating plasma samples of 70 patients before and after surgery. Compared to the preoperative cohort (median, interquartile range: 4.92, 3.52), the KLK6 levels were slightly decreased in the postoperative cohort (median, interquartile range: 4.90, 1.93), though the difference was not significant (*P* = 0.280) (also observed in Figure 3).

## 4. Discussion

The majority of laryngeal SCCs primarily arise without visible changes in the mucosa. Moreover, they may progress from early stages to advanced stages within a few weeks, resulting in late diagnosis and thus a poor treatment outcome [20]. Although excellent biomarkers may improve the early detection and risk assessment of cancer, such biomarkers are compromised by their insufficient sensitivity and specificity and are only applied as auxiliary approaches to assist in clinical decision making. Mass spectrometry-based proteomics is widely applicable in biomarker discovery, and the most common resources employed to identify biomarkers of laryngeal cancer comprise tissues [4–6], blood [7, 8], saliva [9, 10], and cancer cell lines [11, 12]. Given that there are limitations with regard to these proteomic studies, for example, the interference of high-abundance proteins in body fluids, the proteomic analysis of serum-free CM from a primary tissue culture system was applied to establish a laryngeal cancer-derived secretory/releasing protein database, containing 982 CM proteins. To our knowledge, this comprises the largest database with potential laryngeal SCC candidates. Tissue culture conditions have been carefully optimized under serum starvation, marginally reducing cell lysis and thus intracellular contaminants. As outlined above, the culture conditions broadly maintained the integrity of the tissue samples *in vitro* (Figure S1) and have proven to be useful in the enrichment of extracellular proteins [13, 14]. As expected, the secretory/releasing protein database encompassed a significant proportion of extracellular proteins, up to 15.0% of the total CM proteins.

Cancer can be considered an evolutionary process resulting from the coevolution of tumor cells and their microenvironment [22, 23]. This serum-free tissue culture model was similar to the pathological state *in vivo*, and the CM proteins reflected partial characteristics of the tumors and their environment. For instance, the CM proteins were preferentially involved in secreted or membrane protein-associated biological processes, such as cell junction organization, cellular membrane organization, and extracellular matrix organization, and the remodeling of these compartments may generate cell signaling responses, thereby leading to growth, adhesion, and migration [24]. Of particular interest, a set of glycolytic enzymes, including HK1, GPI, ALDOC, TPI, GAPDH, PGK1, PGM1, ENO1, and PKM2, were present in the secretory/releasing protein database, consistent with our previous findings [13, 14], implying the upregulation of glycolysis as an adaptation to the tumor microenvironment. The hallmarks of cancer are recognized as ten biological capabilities acquired during tumor development, including the dysfunction of cellular energetics [25]. Increased glycolysis is one aspect of cancer metabolism that is partly attributed to the dysregulation of glycolytic enzymes and the selective pressure of hypoxia [26, 27].

**Figure 4:**
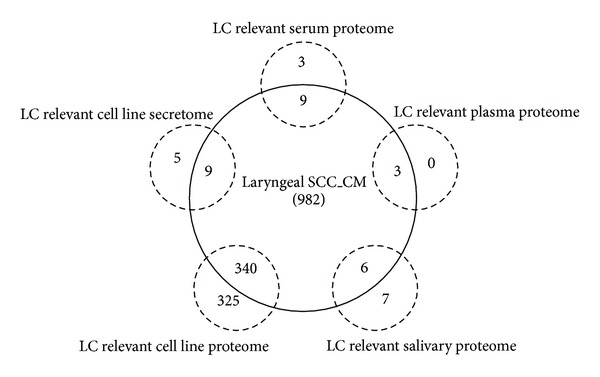
Comparisons of the laryngeal cancer-derived “secretory/releasing proteome” (laryngeal SCC_CM) with five laryngeal cancer-related proteomic studies. The other studies include laryngeal cancer-related serum/plasma proteome [7, 8], salivary proteome [9], and cell line proteome [11, 12].

A small number of proteomic studies have been conducted to mine laryngeal cancer-related markers, though these studies provided limited candidates or insufficient verification. The developed laryngeal cancer-derived secretory/releasing proteome largely overlapped with the laryngeal cancer-derived serum/plasma proteome, saliva proteome, and laryngeal cancer cell line-relevant secretome (Figure 4). Moreover, the proteome in the present study included several laryngeal cancer-associated blood markers identified in previous studies, such as CAT, IL6, IL8, S100A9, PFN1, HSPA1A, MMP2, MMP3, KRT19, SERPINB3, PRDX3, and LGALS3BP. These results verified the quality of the laryngeal cancer-derived “secretory/releasing” protein database in uncovering novel biomarkers for this disease.

It was of paramount importance to minimize the number of candidate biomarkers evaluated for further validation. By applying the aforementioned criteria, the list of candidates was composed of 49 proteins (observed in Table S6). Of these, 24 proteins were also identified within the laryngeal cancer cell line-relevant secretome by Sepiashivili et al., including KLK6, ANXA2, CALR, CLU, TIMP1, TIMP2, and THBS1. The human tissue kallikreins (KLKs) is a family of 15 secreted serine proteases (KLK1-15) with trypsin- or chymotrypsin-like activity and encoded by a cluster of genes tandemly localized on chromosome 19q13.3-4 [28]. KLK6 was initially identified by three different groups, naming it zyme in Alzheimer's disease [29], protease M in breast cancer [30], and neurosin in colon adenocarcinoma [31]. KLK6 participates in other cellular pathways in addition to the degradation of the extracellular matrix, such as inflammation, receptor activation, and apoptosis regulation. The successful application of KLK3 (also known as prostate-specific antigen) for prostate cancer diagnosis and prognosis has led to studies concerning KLK6 as a potential biomarker for cancers. Several reports have shown that KLK6 is a promising biomarker of early diagnosis and unfavorable prognosis in several cancers, including ovarian cancer [32–36], colorectal cancer [37, 38], gastric cancer [39, 40], uterine cancer [41], lung cancer [42], and pancreatic cancer [43]. Given that its expression was negative in larynx tissues but detected in several biological fluids [44], KLK6 was prioritized for the ensuing validation. The KLK6 protein level was found to be elevated in the plasma of laryngeal SCC patients compared to the other two cohorts (i.e., benign cohort and the healthy cohort), whereas its level was not notably different between the other two cohorts (Table 1 and Figure 3). It should be emphasized that this is the first report of KLK6 as a potential blood marker for diagnosing laryngeal SCC. Furthermore, the diagnostic sensitivity and specificity of the plasma KLK6 concentration were examined. As displayed in Table S7 and Figure S3, the sensitivity and specificity were approximately 30% and 80%, respectively, in distinguishing cancer cases from healthy cases, benign cases, or noncancer cases. For classifying cancer cases from noncancer cases, it achieved a sensitivity and specificity of 35.6% and 82.2%, respectively. In addition, this marker detected 40.0% (26/65) of early-stage cancer cases. Despite its poor diagnostic performance as a single biomarker, KLK6 represents a promising candidate for developing a panel with other biomarkers. Correlative studies have already provided evidence that five other protein members of KLK family (KLK4, KLK5, KLK7, KLK8, and KLK10) were abundantly expressed in head and neck squamous cell carcinoma, showing diagnostic value [45]. It will prompt the design of the multianalyte panel stemming from the KLK family to diagnose this disease.

We obtained plasma samples before and after surgery from 70 laryngeal SCC patients and found a nonsignificant decrease in KLK6 concentrations after surgery (Figure 3), implying that plasma KLK6 most likely originates from tumor cells. Additional studies are required to confirm KLK6 as a potential indicator to monitor this disease in a large cohort.

## 5. Conclusions

A laryngeal cancer-derived secretory/releasing protein database was established on the basis of a novel serum-free primary culture model. According to the database, KLK6 was chosen for validation using plasma samples from laryngeal SCC patients, benign patients, and healthy individuals, and the level of KLK6 was shown to be a diagnostic candidate. Further studies are planned to identify additional biomarkers using this high-capacity and reliable database.

## Supplementary Material

Table S1: The clinicopathological data of the laryngeal cancer cases involved in the primary tissue culture.
Table S2: The clinical characteristics of the 149 patients with laryngeal SCC used for ELISA.
Table S3: The clinical characteristics of the 70 patients as the sources of preoperative and postoperative blood samples.
Table S4: The protein identifications in all the CM samples.
Table S5: The list of proteins previously reported in the serum of head and neck cancer patients.
Table S6: Forty-nine candidate biomarkers of laryngeal SCC in the “secretory/releasing” database.
Table S7: The performance of KLK6 in distinguishing cancer cases from controls at selected cutoff points
Figure S1: Representative images of HE-stained sections of normal tissue (A) and matched tumor tissue (B) derived from one laryngeal SCC patient after 0 h and 48 h *in vitro*, respectively. Magnification 200×.
Figure S2: Cellular components significantly enriched using the BiNGO tool, with the Benjamini & Hochberg False Discovery Rate correction for multiple testing and all human annotations as the reference set. The GO terms and their significance (negative log of the *P* value) are separately displayed on the x- and y-axes. “Extracellular region part” and “cell surface” are highlighted in the red box. *P* values less than 0.01 were considered to be statistically significant.
Figure S3: ROC curves with regard to the plasma KLK6 concentration discriminating laryngeal SCC patients from controls. The discriminatory power of the cancer cases from healthy cases, benign cases, and non-cancer cases are shown in A, B, and C, respectively. The values of the area under the ROC curve (AUC) were 0.587 (95% CI: 0.519-0.654; P=0.014) for A, 0.567 (95% CI: 0.501-0.633; P=0.047) for B, and 0.576 (95% CI: 0.517-0.635; P=0.010) for C.

## Figures and Tables

**Table 1:** KLK6 levels in the plasma of laryngeal SCC patients and controls.

Characteristics	KLK6 levels (ng/mL)		*P* value
	Median	Interquartile range	
All individuals	4.76	2.34	
Diagnostic category			
Healthy	4.65	2.32	
Benign	4.65	1.86	0.607*
Cancer	4.90	4.26	0.047^†^
			0.014^‡^
Anatomical region of cancer			
Glottis	4.91	4.06	0.393
Supraglottis	4.88	4.21	
Disease stage			
I + II	5.44	3.60	0.375
III + IV	4.76	4.48	
T stage			
T1	6.06	3.87	0.544
T2	4.98	5.00	
T3	4.98	4.55	
T4	4.60	2.62	
Lymph node status			
pN0	5.15	3.84	0.507
pN+	4.77	4.49	
Tumor differentiation			
Well	4.65	5.09	0.637
Moderate	5.33	4.66	
Poor	4.59	3.85	

KLK6: kallikrein 6.

Comparisons of the plasma KLK6 levels between two independent sample cohorts were assessed by the Mann-Whitney *U* test, and those among more than two independent cohorts were assessed by the Kruskal-Wallis *H* test. *Benign versus health; ^†^cancer versus benign; ^‡^cancer versus health.
