# Supplementary material for: Identification of Human Tissue Kallikrein 6 as a Potential Marker of Laryngeal Cancer Based on the Relevant Secretory/Releasing Protein Database
Source: Dis Markers. 2014 Feb 11;2014:594093. doi: 10.1155/2014/594093 (PMC3942293; doi:10.1155/2014/594093)
Supplement: Supplementary file 1 — Table S1: The clinicopathological data of the laryngeal cancer cases involved in the primary tissue culture. Table S2: The clinical characteristics of the 149 patients with laryngeal SCC used for ELISA. Table S3: The clinical characteristics of the 70 patients as the sources of preoperative and postoperative blood samples. Table S4: The protein identifications in all the CM samples. Table S5: The list of proteins previously reported in the serum of head and neck cancer patients. Table S6: Forty-nine candidate biomarkers of laryngeal SCC in the “secretory/releasing” database. Table S7: The performance of KLK6 in distinguishing cancer cases from controls at selected cutoff points Figure S1: Representative images of HE-stained sections of normal tissue (A) and matched tumor tissue (B) derived from one laryngeal SCC patient after 0 h and 48 h in vitro, respectively. Magnification 200×. Figure S2: Cellular components significantly enriched using the BiNGO tool, with the Benjamini & Hochberg False Discovery Rate correction for multiple testing and all human annotations as the reference set. The GO terms and their significance (negative log of the P value) are separately displayed on the x- and y-axes. “Extracellular region part” and “cell surface” are highlighted in the red box. P values less than 0.01 were considered to be statistically significant. Figure S3: ROC curves with regard to the plasma KLK6 concentration discriminating laryngeal SCC patients from controls. The discriminatory power of the cancer cases from healthy cases, benign cases, and non-cancer cases are shown in A, B, and C, respectively. The values of the area under the ROC curve (AUC) were 0.587 (95% CI: 0.519-0.654; P=0.014) for A, 0.567 (95% CI: 0.501-0.633; P=0.047) for B, and 0.576 (95% CI: 0.517-0.635; P=0.010) for C. [file 594093.f1.pdf]

Table S1. The clinicopathological data of the laryngeal cancer cases involved in the primary tissue culture

Case coding*	Gender	Age at diagnosis	Smoking status	Alcohol status	Histological type	TNM staging†	Tumor differentiation
LC01	male	63	Yes	Yes	Supraglottic squamous cell carcinoma	T4N2M0	Moderate
LC14	male	73	Yes	Yes	Supraglottic squamous cell carcinoma	T3N2M0	Moderate
LC53	male	54	Yes	No	Supraglottic squamous cell carcinoma	T3N2M0	Moderate
LC95	male	50	Yes	Yes	Glottic squmamous cell carcinoma	T3N1M0	Poor

*LC represents laryngeal cancer.

† staged according to Sixth Edition (2002) of the AJCC-UICC TNM Staging System.

Table S2. The clinical characteristics of the 149 patients with laryngeal SCC used for ELISA

Variable	N (%)	Variable	N(%)
Age (years)		Disease stage	
≤55	58 (38.9)	I	33 (22.1)
>55	91 (61.1)	II	32 (21.5)
Median (range)	58 (35-82)	III	41 (27.5)
		IV	41 (27.5)
		Missing	2 (1.3)
Gender		T stage*	
Male	140 (94.0)	T1	33 (21.5)
Female	9 (6.0)	T2	36 (24.2)
		T3	52 (34.9)
		T4	26 (17.4)
		Missing	2 (1.3)
Smoking status		Lymph node status	
Ever	124 (83.2)	pN0	110 (73.8)
Never	11 (7.4)	pN+	37 (24.8)
Missing	14 (9.4)	Missing	2 (1.3)
Alcohol status		Tumor differentiation	
Yes	84 (56.4)	well	34 (22.8)
No	51(34.2)	moderate	78 (52.3)
Missing	14 (9.4)	poor	24 (16.1)
		Missing	13 (8.7)
Anatomical region			
Glottis	82 (55.0)		
Supraglottis	60 (40.3)		
Subglottis	1 (0.7)		
Missing	6 (4.0)		

[*]staged according to Sixth Edition (2002) of the AJCC-UICC TNM Staging System.

Table S3. The clinical characteristics of the 70 patients as the sources of preoperative and postoperative blood samples

Variable	N (%)	Variable	N(%)
Age (years)		T stage*	
≤55	27 (38.6)	T1	11 (15.7)
>55	43 (61.4)	T2	11 (15.7)
Median (range)	59 (40-82)	T3	27 (38.6)
		T4	17 (24.3)
		Missing	4 (5.7)
Gender		Lymph node status	
Male	68 (97.1)	pN0	43 (61.4)
Female	2 (2.9)	pN+	23 (32.9)
		Missing	4 (5.7)
Anatomical region		Tumor differentiation	
Glottis	36 (51.4)	well	14 (20.0)
Supraglottis	28 (40.0)	moderate	41 (58.6)
Subglottis	1 (1.4)	poor	10 (14.3)
Missing	5 (7.1)	Missing	5 (7.1)
Disease			
I	11 (15.7)		
II	11 (15.7)		
III	22 (31.4)		
IV	22 (31.4)		
Missing	4 (5.7)		

*staged according to Sixth Edition (2002) of the AJCC-UICC TNM Staging System.

Table S4. The protein identifications in all the CM samples

Gene name	Description	MW	pI	N1	N14	N53	N95	T1	T14	T53	T95
CLIC1	Chloride intracellular channel protein 1	26905.75	4.552361	1	1	1	1	1	1	1	1
ACTN4	Alpha-actinin-4	104788.5	4.792366	1	1	1	1	1	1	1	1
IDH1	Isocitrate dehydrogenase [NADP] cytoplasmic	46629.51	4.901527	1	1	1	1	1	1	1	1
LDHA	L-lactate dehydrogenase A chain	36665.36	5.837837	1	1	1	1	1	1	1	1
F13A1	Coagulation factor XIII A chain	83214.6	4.660027	1	1	1	1	1	1	1	1
PGK1	Phosphoglycerate kinase 1	44586.13	6.564583	1	1	1	1	1	1	1	1
CA1	Carbonic anhydrase 1	28852.39	5.473824	1	1	1	1	1	1	1	1
	Ig kappa chain V-III region SIE	11767.84	4.285545	1	1	1	1	1	1	1	1
IGKC	Ig kappa chain C region	11601.67	4.599464	1	1	1	1	1	1	1	1
IGHG1	Ig gamma-1 chain C region	36083.17	5.57711	1	1	1	1	1	1	1	1
IGHA1	Ig alpha-1 chain C region	37630.64	5.226448	1	1	1	1	1	1	1	1
IGHA2	Ig alpha-2 chain C region	36503.01	5.122093	1	1	1	1	1	1	1	1
LMNA	Lamin-A/C	74094.71	5.645149	1	1	1	1	1	1	1	1
FGG	Fibrinogen gamma chain	51478.87	4.340981	1	1	1	1	1	1	1	1
ALB	Serum albumin	69321.49	5.051598	1	1	1	1	1	1	1	1
MMP1	Interstitial collagenase	53972.78	4.874825	1	1	1	1	1	1	1	1
ALDOA	Fructose-bisphosphate aldolase A	39395.31	5.413475	1	1	1	1	1	1	1	1
ANXA1	Annexin A1	38689.98	5.054909	1	1	1	1	1	1	1	1
GAPDH	Glyceraldehyde-3-phosphate dehydrogenase	36030.4	5.922539	1	1	1	1	1	1	1	1
HSPB1	Heat shock protein beta-1	22768.49	5.121132	1	1	1	1	1	1	1	1
GSN	Gelsolin	85644.19	4.900246	1	1	1	1	1	1	1	1
ENO1	Alpha-enolase	47139.32	5.217903	1	1	1	1	1	1	1	1
P4HB	Protein disulfide-isomerase	57080.67	4.507607	1	1	1	1	1	1	1	1
ANXA2	Annexin A2	38579.81	4.683418	1	1	1	1	1	1	1	1
CTSL1	Cathepsin L1	37539.67	4.436897	1	1	1	1	1	1	1	1

HSP90AA1	Heat shock protein HSP 90-alpha	84606.68	4.575111	1	1	1	1	1	1	1	1
THBS1	Thrombospondin-1	129299.6	4.356895	1	1	1	1	1	1	1	1
HSPA1A	Heat shock 70 kDa protein 1	70009.04	4.860725	1	1	1	1	1	1	1	1
HSP90AB1	Heat shock protein HSP 90-beta	83212.1	4.599358	1	1	1	1	1	1	1	1
MMP3	Stromelysin-1	53943.29	4.797813	1	1	1	1	1	1	1	1
VIM	Vimentin	53619.08	4.683418	1	1	1	1	1	1	1	1
ANXA5	Annexin A5	35914.4	4.453026	1	1	1	1	1	1	1	1
GSTP1	Glutathione S-transferase P	23341.02	4.118065	1	1	1	1	1	1	1	1
HSPA5	78 kDa glucose-regulated protein	72288.43	4.748039	1	1	1	1	1	1	1	1
HSPA8	Heat shock cognate 71 kDa protein	70854.22	4.825798	1	1	1	1	1	1	1	1
COL6A3	Collagen alpha-3(VI) chain	343452.8	5.171654	1	1	1	1	1	1	1	1
ANXA3	Annexin A3	36352.66	4.764809	1	1	1	1	1	1	1	1
EEF2	Elongation factor 2	95276.95	5.217476	1	1	1	1	1	1	1	1
LCP1	Plastin-2	70244.84	4.675407	1	1	1	1	1	1	1	1
HSP90B1	Endoplasmin	92411.34	4.475349	1	1	1	1	1	1	1	1
EZR	Ezrin	69369.74	5.085564	1	1	1	1	1	1	1	1
DSP	Desmoplakin	331568.7	5.13694	1	1	1	1	1	1	1	1
VCL	Vinculin	123721.8	5.229652	1	1	1	1	1	1	1	1
PGAM1	Phosphoglycerate mutase 1	28785.83	5.327278	1	1	1	1	1	1	1	1
SDC1	Syndecan-1	32456.83	4.365013	1	1	1	1	1	1	1	1
PPIB	Peptidyl-prolyl cis-trans isomerase B	23727.53	9.024773	1	1	1	1	1	1	1	1
PSMA4	Proteasome subunit alpha type-4	29465.18	4.961555	1	1	1	1	1	1	1	1
MSN	Moesin	67777.79	5.292992	1	1	1	1	1	1	1	1
TKT	Transketolase	67834.72	5.550835	1	1	1	1	1	1	1	1
PRDX6	Peroxiredoxin-6	25019.19	5.206581	1	1	1	1	1	1	1	1
SERPINB1	Leukocyte elastase inhibitor	42714.7	4.986763	1	1	1	1	1	1	1	1
YWHAB	14-3-3 protein beta/alpha	28064.83	4.288322	1	1	1	1	1	1	1	1
SFN	14-3-3 protein sigma	27756.69	4.292915	1	1	1	1	1	1	1	1

HSPA4	Heat shock 70 kDa protein 4	94271.24	4.676582	1	1	1	1	1	1	1	1
TALDO1	Transaldolase	37516.46	5.158089	1	1	1	1	1	1	1	1
GDI2	Rab GDP dissociation inhibitor beta	50630.88	4.491585	1	1	1	1	1	1	1	1
LUM	Lumican	38404.79	4.608223	1	1	1	1	1	1	1	1
PGD	6-phosphogluconate dehydrogenase, decarboxylating	53105.95	5.222282	1	1	1	1	1	1	1	1
VCP	Transitional endoplasmic reticulum ATPase	89265.72	4.834129	1	1	1	1	1	1	1	1
NPEPPS	Puromycin-sensitive aminopeptidase	103210.7	4.693245	1	1	1	1	1	1	1	1
TPI1	Triosephosphate isomerase	26652.74	5.462929	1	1	1	1	1	1	1	1
ACTR3	Actin-related protein 3	47340.98	4.616768	1	1	1	1	1	1	1	1
YWHAE	14-3-3 protein epsilon	29155.42	4.193367	1	1	1	1	1	1	1	1
HIST1H4A	Histone H4	11360.38	11.33447	1	1	1	1	1	1	1	1
PPIA	Peptidyl-prolyl cis-trans isomerase A	18000.88	6.427971	1	1	1	1	1	1	1	1
RPS27A	Ubiquitin	8559.617	5.727928	1	1	1	1	1	1	1	1
YWHAZ	14-3-3 protein zeta/delta	27727.73	4.263756	1	1	1	1	1	1	1	1
HBB	Hemoglobin subunit beta	15988.29	6.101128	1	1	1	1	1	1	1	1
HBA1	Hemoglobin subunit alpha	15247.93	6.783974	1	1	1	1	1	1	1	1
GSTO1	Glutathione S-transferase omega-1	27548.04	4.847694	1	1	1	1	1	1	1	1
CLTC	Clathrin heavy chain 1	191492.5	4.581413	1	1	1	1	1	1	1	1
PSME1	Proteasome activator complex subunit 1	28705.01	5.041557	1	1	1	1	1	1	1	1
PRDX1	Peroxiredoxin-1	22096.28	5.780907	1	1	1	1	1	1	1	1
TRAP1	Heat shock protein 75 kDa, mitochondrial	80059.69	5.454491	1	1	1	1	1	1	1	1
TGFBI	Transforming growth factor-beta-induced protein ig-h3	74633.98	5.535027	1	1	1	1	1	1	1	1
GLOD4	Glyoxalase domain-containing protein 4	34771.25	4.656075	1	1	1	1	1	1	1	1
PSMA7	Proteasome subunit alpha type-7	27869.61	5.300041	1	1	1	1	1	1	1	0
ALDH1A1	Retinal dehydrogenase 1	54826.95	4.90078	0	1	1	1	1	1	1	1
NP	Purine nucleoside phosphorylase	32097.16	5.142921	1	0	1	1	1	1	1	1
IGLC1	Ig lambda chain C regions	11229.53	4.891914	1	1	1	1	1	1	1	0

IGHG3	Ig gamma-3 chain C region	41260.36	5.79351	1	0	1	1	1	1	1	1
IGHG4	Ig gamma-4 chain C region	35917.87	4.997871	0	1	1	1	1	1	1	1
FGB	Fibrinogen beta chain	55892.26	5.143883	1	0	1	1	1	1	1	1
FN1	Fibronectin	262442.1	4.429314	1	1	1	1	1	1	1	0
TF	Serotransferrin	76999.61	5.106071	1	1	1	1	0	1	1	1
LTF	Lactotransferrin	78131.91	5.604774	0	1	1	1	1	1	1	1
FTL	Ferritin light chain	20007.1	4.786598	0	1	1	1	1	1	1	1
CSTB	Cystatin-B	11132.59	5.887718	1	1	1	1	0	1	1	1
S100A8	Protein S100-A8	10827.65	5.460152	1	1	1	1	0	1	1	1
SERPINE1	Plasminogen activator inhibitor 1	45031.03	5.595055	1	1	1	1	1	1	0	1
S100A9	Protein S100-A9	13233.51	5.553505	1	1	1	1	1	1	1	0
GPI	Glucose-6-phosphate isomerase	63107.24	6.022835	0	1	1	1	1	1	1	1
LDHB	L-lactate dehydrogenase B chain	36615.14	4.992317	0	1	1	1	1	1	1	1
CAPN1	Calpain-1 catalytic subunit	81838.19	4.74398	1	1	1	1	0	1	1	1
DCN	Decorin	39721.84	6.312187	0	1	1	1	1	1	1	1
PFN1	Profilin-1	15044.56	5.174644	0	1	1	1	1	1	1	1
MMP2	72 kDa type IV collagenase	73834.71	4.356895	1	1	1	1	1	1	1	0
LAMC1	Laminin subunit gamma-1	177488.5	4.56913	0	1	1	1	1	1	1	1
ACTN1	Alpha-actinin-1	102992.6	4.74868	0	1	1	1	1	1	1	1
CDH1	Epithelial cadherin	97395.86	4.200951	1	1	1	1	0	1	1	1
PDIA4	Protein disulfide-isomerase A4	72886.97	4.442238	0	1	1	1	1	1	1	1
AKR1A1	Alcohol dehydrogenase [NADP+]	36549.86	4.850578	0	1	1	1	1	1	1	1
PKM2	Pyruvate kinase isozymes M1/M2	57900.02	6.00959	1	1	1	1	0	1	1	1
GOT1	Aspartate aminotransferase, cytoplasmic	46218.53	5.119316	0	1	1	1	1	1	1	1
LGALS3	Galectin-3	26136.05	4.393425	0	1	1	1	1	1	1	1
TYMP	Thymidine phosphorylase	49924.17	5.275261	0	1	1	1	1	1	1	1
OGN	Mimecan	33900.88	4.623177	1	1	1	1	1	1	0	1
UBA1	Ubiquitin-like modifier-activating enzyme 1	117774.3	4.740456	0	1	1	1	1	1	1	1

TNC	Tenascin	240713.2	4.368538	0	1	1	1	1	1	1	1
EEF1G	Elongation factor 1-gamma	50087.14	4.988045	0	1	1	1	1	1	1	1
CALR	Calreticulin	48111.82	4.054939	0	1	1	1	1	1	1	1
PEBP1	Phosphatidylethanolamine-binding protein 1	21043.67	4.87632	0	1	1	1	1	1	1	1
PDIA3	Protein disulfide-isomerase A3	56746.75	4.860298	0	1	1	1	1	1	1	1
S100A11	Protein S100-A11	11732.82	4.989113	1	1	1	1	1	1	1	0
PRDX2	Peroxiredoxin-2	21878.24	4.682991	0	1	1	1	1	1	1	1
SERPINB5	Serpin B5	42111.45	5.245461	1	0	1	1	1	1	1	1
COL18A1	Collagen alpha-1(XVIII) chain	178077.1	5.186501	0	1	1	1	1	1	1	1
CAPG	Macrophage-capping protein	38493.51	5.059502	1	1	1	1	1	1	0	1
ADH7	Alcohol dehydrogenase class 4 mu/sigma chain	39979.54	6.657402	1	1	1	1	1	1	1	0
MDH1	Malate dehydrogenase, cytoplasmic	36403.02	5.27227	0	1	1	1	1	1	1	1
MDH2	Malate dehydrogenase, mitochondrial	35480.73	8.062401	0	1	1	1	1	1	1	1
IQGAP1	Ras GTPase-activating-like protein IQGAP1	189133.8	4.870659	0	1	1	1	1	1	1	1
CAPZB	F-actin-capping protein subunit beta	31330.77	4.582161	1	1	0	1	1	1	1	1
ARHGDIA	Rho GDP-dissociation inhibitor 1	23192.7	4.486351	0	1	1	1	1	1	1	1
CAPZA1	F-actin-capping protein subunit alpha-1	32902.33	4.721336	0	1	1	1	1	1	1	1
BLVRA	Biliverdin reductase A	33407.33	5.342125	1	1	1	1	0	1	1	1
PSMA6	Proteasome subunit alpha type-6	27381.81	4.777306	1	0	1	1	1	1	1	1
YWHAG	14-3-3 protein gamma	28284.91	4.203407	0	1	1	1	1	1	1	1
LCN2	Neutrophil gelatinase-associated lipocalin	22573.72	5.108849	1	1	1	1	1	1	1	0
HSPG2	Basement membrane-specific heparan sulfate proteoglycan core protein	468500.5	5.185219	0	1	1	1	1	1	1	1
COL14A1	Collagen alpha-1(XIV) chain	193394.5	4.537407	1	1	1	1	1	1	0	1
TGM3	Protein-glutamine gamma-glutamyltransferase E	76709.71	4.84182	1	1	1	1	0	1	1	1
ILF2	Interleukin enhancer-binding factor 2	43035.19	4.836266	0	1	1	1	1	1	1	1
NACA	Nascent polypeptide-associated complex subunit alpha	23369.71	4.424934	0	1	1	1	1	1	1	1

BLMH	Bleomycin hydrolase	52528.02	4.94265	0	1	1	1	1	1	1	1
PDIA6	Protein disulfide-isomerase A6	48091.26	4.556206	0	1	1	1	1	1	1	1
PLEC1	Plectin-1	531466	5.145699	0	1	1	1	1	1	1	1
ECM1	Extracellular matrix protein 1	60635.27	5.563118	1	1	1	1	1	1	1	0
PGM2	Phosphoglucomutase-2	68240.39	5.017097	0	1	1	1	1	1	1	1
FAM49B	Protein FAM49B	36724.65	4.897896	0	1	1	1	1	1	1	1
DPP3	Dipeptidyl-peptidase 3	82537.8	4.377724	0	1	1	1	1	1	1	1
SERPINB13	Serpin B13	44248.15	4.864357	1	1	1	1	1	1	1	0
A2ML1	Alpha-2-macroglobulin-like protein 1	161001.1	4.369499	1	1	1	1	1	1	0	0
KLK10	Kallikrein-10	30118.31	7.076103	1	1	1	1	1	1	0	0
FLNB	Filamin-B	278021	4.637276	0	1	1	0	1	1	1	1
PGLS	6-phosphogluconolactonase	27529.55	5.247276	0	1	1	0	1	1	1	1
SOD1	Superoxide dismutase [Cu-Zn]	15925.9	5.696098	0	1	1	1	1	1	1	0
GOT2	Aspartate aminotransferase, mitochondrial	47445.27	6.602287	0	1	1	1	0	1	1	1
TIMP1	Metalloproteinase inhibitor 1	23155.55	6.278862	1	1	1	1	1	1	0	0
IGHG2	Ig gamma-2 chain C region	35877.76	5.371925	1	1	1	1	1	0	0	1
KRT6A	Keratin, type II cytoskeletal 6A	60008.27	5.035789	0	1	1	1	0	1	1	1
FGA	Fibrinogen alpha chain	94914.41	4.889244	0	0	1	1	1	1	1	1
CAT	Catalase	59718.75	5.311577	0	1	1	1	0	1	1	1
SOD2	Superoxide dismutase [Mn], mitochondrial	24706.55	5.605522	0	1	1	1	1	1	1	0
KRT1	Keratin, type II cytoskeletal 1	65977.99	4.738533	0	1	1	1	0	1	1	1
SERPINB2	Plasminogen activator inhibitor 2	46566.15	4.655327	1	1	1	1	1	1	0	0
RPLP0	60S acidic ribosomal protein P0	34251.8	5.000221	0	0	1	1	1	1	1	1
ATP5B	ATP synthase subunit beta, mitochondrial	56524.6	4.693993	1	1	1	1	0	1	0	1
HIST1H2BJ	Histone H2B type 1-J	13895.58	10.16573	0	1	1	1	0	1	1	1
IVL	Involucrin	68427.18	4.596581	1	1	1	1	0	1	1	0
ANXA4	Annexin A4	35860.12	4.697197	1	0	0	1	1	1	1	1
LTA4H	Leukotriene A-4 hydrolase	69241.24	4.774529	0	1	1	1	1	1	0	1

ALDOC	Fructose-bisphosphate aldolase C	39431.24	4.916267	0	0	1	1	1	1	1	1
CKMT1A	Creatine kinase, ubiquitous mitochondrial	47007.21	5.930229	0	1	1	1	0	1	1	1
PEPD	Xaa-Pro dipeptidase	54512.91	4.890419	0	1	1	1	0	1	1	1
XRCC6	ATP-dependent DNA helicase 2 subunit 1	69799.05	5.020515	0	1	1	1	1	1	1	0
RNH1	Ribonuclease inhibitor	49941.09	4.610466	0	1	1	1	1	1	1	0
KRT10	Keratin, type I cytoskeletal 10	59474.91	4.259911	0	1	1	1	0	1	1	1
KRT5	Keratin, type II cytoskeletal 5	62339.98	5.008018	0	1	1	1	0	1	1	1
PLS3	Plastin-3	70391.1	4.874504	0	1	1	1	0	1	1	1
APEH	Acylamino-acid-releasing enzyme	81172.61	4.609505	0	1	0	1	1	1	1	1
ANXA8	Annexin A8	36857.65	4.651482	0	1	1	1	0	1	1	1
ENO3	Beta-enolase	46957.32	5.343193	0	1	1	0	1	1	1	1
MIF	Macrophage migration inhibitory factor	12468.22	4.633858	0	1	1	1	0	1	1	1
MMP9	Matrix metalloproteinase-9	78349.31	4.545311	1	0	1	1	1	0	1	1
NME1	Nucleoside diphosphate kinase A	17137.67	5.081398	0	0	1	1	1	1	1	1
HSPA6	Heat shock 70 kDa protein 6	70984.22	5.091545	1	1	1	0	1	1	0	1
KRT4	Keratin, type II cytoskeletal 4	57249.83	5.151466	0	1	1	1	0	1	1	1
PSMB1	Proteasome subunit beta type-1	26472.37	4.968071	0	1	1	1	1	1	1	0
TNXB	Tenascin-X	464164.8	4.624565	1	1	1	1	1	1	0	0
WARS	Tryptophanyl-tRNA synthetase, cytoplasmic	53131.61	4.826973	0	1	1	0	1	1	1	1
AHCY	Adenosylhomocysteinase	47685.2	4.828148	0	1	1	0	1	1	1	1
CFL1	Cofilin-1	18490.66	5.187996	0	1	1	1	1	1	1	0
PSMA1	Proteasome subunit alpha type-1	29536.86	5.158836	1	0	0	1	1	1	1	1
SERPINB3	Serpin B3	44536.54	5.124123	0	1	1	1	0	1	1	1
BLVRB	Flavin reductase	22105.43	5.774071	0	1	1	1	0	1	1	1
PRDX3	Thioredoxin-dependent peroxide reductase, mitochondrial	27675.18	5.954155	0	1	0	1	1	1	1	1
ALDH3A1	Aldehyde dehydrogenase, dimeric NADP-preferring	50346.85	4.791298	1	1	1	1	0	0	1	1
HSPA1L	Heat shock 70 kDa protein 1L	70331.32	4.932076	0	1	1	0	1	1	1	1

MYH9	Myosin-9	226391.6	5.119423	0	1	1	1	0	1	1	1
NAMPT	Nicotinamide phosphoribosyltransferase	55486.59	4.75605	0	1	1	1	1	1	0	1
PSMB2	Proteasome subunit beta type-2	22821.68	4.462959	0	1	1	1	0	1	1	1
ARPC4	Actin-related protein 2/3 complex subunit 4	19654.3	6.532112	1	0	1	1	1	0	1	1
EEF1A1	Elongation factor 1-alpha 1	50109.11	6.807365	1	1	1	1	0	1	0	1
HIST1H3A	Histone H3.1	15394.48	11.0935	1	1	1	1	0	1	1	0
CAP1	Adenylyl cyclase-associated protein 1	51822.7	5.734337	0	0	1	1	1	1	1	1
MTAP	S-methyl-5'-thioadenosine phosphorylase	31215.84	5.859306	0	1	1	1	1	1	1	0
SPTAN1	Spectrin alpha chain, brain	284364.2	4.942009	0	0	1	1	1	1	1	1
POSTN	Periostin	93255.27	5.506935	0	0	1	1	1	1	1	1
RUNDC3A	RUN domain-containing protein 3A	49715.79	4.658424	1	0	1	1	1	1	0	1
NCCRP1	Non-specific cytotoxic cell receptor protein 1 homolog	30827.88	5.516975	1	1	1	1	0	0	1	1
CAND1	Cullin-associated NEDD8-dissociated protein 1	136288.7	4.811378	0	0	1	1	1	1	1	1
TXNDC5	Thioredoxin domain-containing protein 5	47598.66	4.884438	1	0	0	1	1	1	1	1
PACAP	Proapoptotic caspase adapter protein	20681.23	4.687904	0	1	0	1	1	1	1	1
CNDP2	Cytosolic non-specific dipeptidase	52844.91	4.713966	0	1	0	1	1	1	1	1
PARK7	Protein DJ-1	19878.49	5.47126	0	1	1	1	1	1	1	0
ERAP1	Endoplasmic reticulum aminopeptidase 1	107166.3	4.958778	0	1	1	0	1	1	1	1
NUDT5	ADP-sugar pyrophosphatase	24312.24	4.47065	0	1	0	1	1	1	1	1
PSME2	Proteasome activator complex subunit 2	27344.31	4.78788	0	0	1	1	1	1	1	1
QSOX1	Sulfhydryl oxidase 1	82525.5	6.878609	0	1	1	1	0	1	0	1
GGCT	Gamma-glutamylcyclotransferase	20994.33	4.403999	0	0	1	0	1	1	1	1
CLSTN1	Calsyntenin-1	109723.7	4.482399	0	1	1	1	1	1	0	0
GSR	Glutathione reductase, mitochondrial	56220.95	6.251732	0	1	0	1	1	1	1	0
CA2	Carbonic anhydrase 2	29227.92	5.69268	0	0	1	1	1	1	1	0
SERPINA1	Alpha-1-antitrypsin	46707.02	5.012291	1	0	1	1	0	1	1	0
A2M	Alpha-2-macroglobulin	163174.9	4.784035	1	0	1	1	1	1	0	0

C3	Complement C3	187029.9	4.842781	1	1	1	1	0	1	0	0
IGJ	Immunoglobulin J chain	15584.55	4.181938	0	1	1	1	1	1	0	0
KRT14	Keratin, type I cytoskeletal 14	51589.44	4.595619	0	1	1	1	0	0	1	1
VWF	von Willebrand factor	309092	5.32	1	1	1	1	1	0	0	0
RPN2	Dolichyl-diphosphooligosaccharide--protein glycosyltransferase subunit 2	69240.97	4.670387	0	0	0	1	1	1	1	1
MPO	Myeloperoxidase	83814.84	6.447517	0	0	0	1	1	1	1	1
IL6	Interleukin-6	23703.14	5.310188	0	1	1	1	1	1	0	0
CTSD	Cathepsin D	44523.63	4.373131	0	1	1	1	1	1	0	0
SOD3	Extracellular superoxide dismutase [Cu-Zn]	25834.67	5.694817	1	1	1	1	0	1	0	0
KRT19	Keratin, type I cytoskeletal 19	44065.11	4.511452	0	1	0	1	0	1	1	1
HNRNPA1	Heterogeneous nuclear ribonucleoprotein A1	38822.13	5.178169	0	0	1	1	0	1	1	1
HIST1H2A G	Histone H2A type 1	14082.95	10.83843	0	1	1	1	0	1	1	0
COL6A1	Collagen alpha-1(VI) chain	108462	4.665581	0	1	1	1	1	1	0	0
XRCC5	ATP-dependent DNA helicase 2 subunit 2	82652.27	5.061638	1	0	1	0	1	0	1	1
KRT13	Keratin, type I cytoskeletal 13	49555.4	4.462105	0	1	1	1	0	0	1	1
JUP	Junction plakoglobin	81692.73	4.798454	1	1	1	1	0	0	1	0
CBR1	Carbonyl reductase [NADPH] 1	30355.86	7.011162	0	1	1	1	0	1	1	0
FAH	Fumarylacetoacetase	46344.21	5.133095	0	0	1	1	0	1	1	1
KRT15	Keratin, type I cytoskeletal 15	49167.06	4.410622	0	1	1	1	0	1	1	0
FLNA	Filamin-A	280563.9	4.752205	0	1	0	1	0	1	1	1
BGN	Biglycan	41627.54	5.454491	0	0	1	0	1	1	1	1
GPX3	Glutathione peroxidase 3	25499.03	4.985054	1	0	1	1	0	1	0	1
NME2	Nucleoside diphosphate kinase B	17286.94	6.09568	0	1	1	1	1	1	0	0
PSMA2	Proteasome subunit alpha type-2	25882.27	4.520531	0	1	0	1	0	1	1	1
PTX3	Pentraxin-related protein PTX3	41992.83	4.9	1	1	1	1	1	0	0	0
YWHAQ	14-3-3 protein theta	27746.77	4.211098	0	1	1	0	0	1	1	1
PSMB4	Proteasome subunit beta type-4	29185.48	4.22007	0	1	0	1	1	1	0	1

S100A2	Protein S100-A2	11109.36	4.454735	0	1	1	0	1	1	1	0
SDC4	Syndecan-4	21628.03	4.139854	0	1	1	1	0	1	1	0
KRT9	Keratin, type I cytoskeletal 9	62091.91	4.278709	0	1	1	1	0	0	1	1
CHI3L1	Chitinase-3-like protein 1	42598.4	5.187675	0	1	1	1	1	1	0	0
TAGLN2	Transgelin-2	22377.17	5.07296	0	1	1	0	1	1	1	0
SERPINB9	Serpin B9	42376.31	5.088234	0	1	1	0	1	1	0	1
ST13	Hsc70-interacting protein	41305.48	4.916695	0	1	1	0	0	1	1	1
AK2	Adenylate kinase 2, mitochondrial	26460.77	5.797142	1	0	0	1	1	1	1	0
EIF3B	Eukaryotic translation initiation factor 3 subunit B	92423.82	4.590599	0	1	1	0	1	1	1	0
ACTB	Actin, cytoplasmic 1	41709.73	4.503334	0	1	0	1	0	1	1	1
ACTR2	Actin-related protein 2	44732.23	4.871727	0	0	1	1	0	1	1	1
RPL10A	60S ribosomal protein L10a	24815.53	9.763481	1	1	0	1	1	1	0	0
EIF5A	Eukaryotic translation initiation factor 5A-1	16821.4	4.749962	0	1	1	1	0	1	1	0
TUBA1B	Tubulin alpha-1B chain	50119.6	4.335213	0	1	1	0	0	1	1	1
FABP5	Fatty acid-binding protein, epidermal	15154.55	5.536949	0	0	1	1	0	1	1	1
KRT17	Keratin, type I cytoskeletal 17	48076.04	4.523415	0	1	0	1	0	1	1	1
CRYZ	Quinone oxidoreductase	35184.54	5.45385	0	1	0	0	1	1	1	1
LGALS3BP	Galectin-3-binding protein	65289.28	4.341515	0	1	1	1	1	1	0	0
HNRNPD	Heterogeneous nuclear ribonucleoprotein D0	38410.3	4.626381	0	0	1	0	1	1	1	1
KPNB1	Importin subunit beta-1	97108.01	4.3256	0	1	1	1	0	1	0	1
SF3B3	Splicing factor 3B subunit 3	135491.7	4.555138	0	0	0	1	1	1	1	1
ERO1L	ERO1-like protein alpha	54358.05	4.733299	0	1	0	1	0	1	1	1
FAM129B	Niban-like protein 1	82630.93	4.888176	1	1	1	1	0	1	0	0
VAT1	Synaptic vesicle membrane protein VAT-1 homolog	41893.42	4.937096	0	1	1	1	1	1	0	0
UGCGL1	UDP-glucose:glycoprotein glucosyltransferase 1	177078	4.577568	0	0	0	1	1	1	1	1
PIR	Pirin	32093.26	5.673882	0	0	1	0	1	1	0	1

ARPC2	Actin-related protein 2/3 complex subunit 2	34311.48	5.300682	0	0	1	1	0	0	1	1
ARPC3	Actin-related protein 2/3 complex subunit 3	20533.37	8.78	1	0	1	1	1	0	0	0
AKR1B10	Aldo-keto reductase family 1 member B10	35997.82	5.344261	0	0	1	0	1	1	0	1
CUTA	Protein CutA	19104.11	4.660774	0	1	0	0	0	1	1	1
WDR1	WD repeat-containing protein 1	66151.87	4.816399	0	1	1	0	0	1	1	0
CS	Citrate synthase, mitochondrial	51679.51	5.411125	0	1	0	0	1	1	0	1
GLUD1	Glutamate dehydrogenase 1, mitochondrial	61359.19	5.222816	0	1	0	1	0	1	0	1
ASS1	Argininosuccinate synthase	46500.96	5.074883	0	1	1	0	0	1	1	0
	Ig lambda chain V-IV region Bau	11298.43	3.158363	0	1	1	1	0	1	0	0
	Ig heavy chain V-III region BRO	13218.4	0.995857	0	0	1	1	0	1	0	1
PIGR	Polymeric immunoglobulin receptor	83231.65	4.657249	0	1	1	0	0	1	1	0
HBD	Hemoglobin subunit delta	16045.29	6.356728	0	0	1	1	0	1	1	0
FTH1	Ferritin heavy chain	21212.29	4.575539	0	1	0	1	1	1	0	0
APOB	Apolipoprotein B-100	515240.8	5.101158	1	1	1	1	0	0	0	0
HIST1H2A B	Histone H2A type 1-B/E	14126.95	11.00004	0	1	0	0	0	1	1	1
KRT8	Keratin, type II cytoskeletal 8	53671.13	4.774956	0	1	0	0	0	1	1	1
PTMA	Prothymosin alpha	12195.97	3.686119	0	0	1	0	0	1	1	1
CEACAM5	Carcinoembryonic antigen-related cell adhesion molecule 5	76748.17	5.43	1	1	0	1	1	0	0	0
LAMB1	Laminin subunit beta-1	197936.6	4.416603	0	1	0	0	1	1	1	0
ANXA6	Annexin A6	75825.58	4.739815	0	0	1	1	1	1	0	0
KRT16	Keratin, type I cytoskeletal 16	51236.2	4.512733	0	1	1	1	0	0	1	0
PTPRF	Receptor-type tyr ine-protein ph phatase F	212744.5	5.92	1	1	1	0	1	0	0	0
ALAD	Delta-aminolevulinic acid dehydratase	36271.41	4.9795	0	0	1	1	1	1	0	0
IL1RN	Interleukin-1 receptor antagonist protein	20041.96	5.23542	0	1	0	1	0	1	1	0
AZU1	Azurocidin	26868.65	9.405235	0	0	0	1	1	1	0	1
APEX1	DNA-(apurinic or apyrimidinic site) lyase	35532.17	5.294273	0	0	0	0	1	1	1	1
PSMB8	Proteasome subunit beta type-8	30334.61	4.59124	0	0	1	1	0	1	1	0

PRDX5	Peroxiredoxin-5, mitochondrial	22012.49	8.629143	0	1	1	1	0	1	0	0
DSG3	Desmoglein-3	107435.4	4.38456	0	1	1	1	0	1	0	0
RDX	Radixin	68521.39	5.293953	0	1	1	0	0	1	0	1
KRT2	Keratin, type II cytoskeletal 2 epidermal	65825.39	5.12252	0	0	1	1	0	0	1	1
GARS	Glycyl-tRNA synthetase	83086.59	5.21801	0	1	1	0	1	1	0	0
GNPDA1	Glucosamine-6-phosphate isomerase 1	32647.58	5.024361	1	1	0	0	1	0	0	1
CAPZA2	F-actin-capping protein subunit alpha-2	32928.61	4.799736	1	0	0	1	0	1	0	1
PREP	Prolyl endopeptidase	80648.03	4.353371	0	0	1	0	0	1	1	1
HDGF	Hepatoma-derived growth factor	26771.87	4.539757	0	1	0	0	1	1	1	0
ARHGDIB	Rho GDP-dissociation inhibitor 2	22973.6	4.602242	0	0	1	1	0	1	1	0
UBE2K	Ubiquitin-conjugating enzyme E2 K	22392.57	4.663872	0	0	1	0	0	1	1	1
LYZ	Lysozyme C	16526.28	8.480034	0	1	0	1	0	1	0	1
AP2B1	AP-2 complex subunit beta-1	104486	4.566353	0	0	1	1	0	0	1	1
ACTC1	Actin, alpha cardiac muscle 1	41991.88	5.23	1	0	1	1	1	0	0	0
CYCS	Cytochrome c	11741.13	9.077003	0	1	1	0	1	1	0	0
PITPNA	Phosphatidylinositol transfer protein alpha isoform	31786.02	4.892342	0	1	0	1	0	1	1	0
SELENBP1	Selenium-binding protein 1	52357.62	4.791405	0	1	1	0	1	0	0	1
PLS1	Plastin-1	70209.26	4.760429	1	0	1	1	0	1	0	0
GANAB	Neutral alpha-glucosidase AB	106806.6	4.713112	0	1	1	0	1	1	0	0
MVP	Major vault protein	99265.93	4.923424	0	0	0	1	1	1	1	0
CLCA4	Calcium-activated chloride channel regulator 4	101219.1	4.662056	1	1	1	1	0	0	0	0
SEPT2	Septin-2	41461.25	5.379829	0	0	1	1	0	1	0	1
RSU1	Ras suppressor protein 1	31520.69	5.727928	0	1	0	1	0	1	1	0
TSN	Translin	26166.78	5.041878	0	0	0	1	0	1	1	1
DDB1	DNA damage-binding protein 1	126887.3	4.570625	0	0	0	0	1	1	1	1
HADH	Hydroxyacyl-coenzyme A dehydrogenase, mitochondrial	34255.9	6.462471	1	0	0	0	1	1	0	1

UGP2	UTP--glucose-1-phosphate uridylyltransferase	56904.72	5.48568	0	1	0	0	1	1	0	1
PDCD6IP	Programmed cell death 6-interacting protein	95963.12	4.716743	1	1	1	1	0	0	0	0
HSPH1	Heat shock protein 105 kDa	96803.66	4.85186	0	0	1	0	1	1	1	0
ISOC1	Isochorismatase domain-containing protein 1	32216.12	5.593132	0	1	1	0	1	1	0	0
ABHD14B	Abhydrolase domain-containing protein 14B	22331.56	5.039101	0	1	1	1	0	1	0	0
VPS35	Vacuolar protein sorting-associated protein 35	91648.93	4.657997	0	1	1	1	0	1	0	0
LGMN	Legumain	49379.41	4.704353	0	0	1	1	0	0	1	1
COL12A1	Collagen alpha-1(XII) chain	332940.5	4.503014	0	0	0	0	1	1	1	1
API5	Apoptosis inhibitor 5	57525.14	4.967003	0	0	0	0	1	1	1	1
XPNPEP1	Xaa-Pro aminopeptidase 1	69873.24	4.667397	0	1	0	1	0	1	0	1
SPRR3	Small proline-rich protein 3	18142.23	8.076607	1	1	1	1	0	0	0	0
CRNN	Cornulin	53501.64	5.420738	1	1	1	1	0	0	0	0
USP25	Ubiquitin carboxyl-terminal hydrolase 25	125671.3	4.69976	0	1	1	1	0	1	0	0
NAGK	N-acetyl-D-glucosamine kinase	37351.92	4.818855	0	1	0	1	0	1	0	1
CORO1C	Coronin-1C	53215.06	5.46389	0	0	0	0	1	1	1	1
TLN1	Talin-1	269599	5.272804	0	0	0	1	1	1	0	1
HEBP2	Heme-binding protein 2	22861.19	4.129387	0	1	1	0	0	1	1	0
PSAT1	Phosphoserine aminotransferase	40396.81	4.957069	0	0	0	0	1	1	1	1
DDX39	ATP-dependent RNA helicase DDX39	49097.95	4.916267	0	1	0	0	1	0	0	1
IPO5	Importin-5	123549.9	4.475243	0	0	0	0	1	1	1	0
AGRN	Agrin	214706.4	5.31414	0	0	1	0	1	0	1	0
TMPRSS11D	Transmembrane protease, serine 11D	46234.37	5.581169	1	0	1	1	0	0	0	0
PPL	Periplakin	204525.9	5.056297	1	1	0	1	0	0	0	0
SYNCRIP	Heterogeneous nuclear ribonucleoprotein Q	69559.6	4.835304	0	0	0	0	0	1	1	1
M6PRBP1	Mannose-6-phosphate receptor-binding protein 1	47017.91	4.943398	0	1	1	0	0	0	0	1
USO1	General vesicular transport factor p115	107828.1	4.85	1	0	1	0	1	0	0	0
CLIC3	Chloride intracellular channel protein 3	26631.71	5.053734	0	1	1	1	0	0	0	0

AGR2	Anterior gradient protein 2 homolog	19966.62	5.541542	0	1	0	0	1	1	0	0
CP	Ceruloplasmin	122127.6	4.370674	0	0	0	1	1	1	0	0
HPRT1	Hypoxanthine-guanine phosphoribosyltransferase	24563.6	4.616341	0	0	0	0	1	1	1	0
HP	Haptoglobin	45176.56	4.657356	1	0	1	1	0	0	0	0
CFB	Complement factor B	85478.52	4.870232	0	0	1	1	0	1	0	0
SERPINC1	Antithrombin-III	52568.86	5.065483	1	0	1	1	0	0	0	0
SERPINA3	Alpha-1-antichymotrypsin	47620.54	4.809563	1	1	0	0	0	1	0	0
CSTA	Cystatin-A	10999.66	4.36544	1	1	1	0	0	0	0	0
	Ig kappa chain V-II region Cum	12668.31	3.851677	0	0	1	1	0	1	0	0
IGHM	Ig mu chain C region	49275.53	5.167809	1	0	1	1	0	0	0	0
SLPI	Antileukoproteinase	14315.91	8.963142	0	0	1	1	0	1	0	0
VTN	Vitronectin	54271.17	4.506111	0	0	1	1	0	0	1	0
SERPING1	Plasma protease C1 inhibitor	55119.39	6.09	1	0	1	0	1	0	0	0
TPM3	Tropomyosin alpha-3 chain	32798.74	4.540398	0	0	0	1	1	0	0	1
SERPINE2	Glia-derived nexin	43974.2	8.915504	0	0	1	0	1	1	0	0
FH	Fumarate hydratase, mitochondrial	54602.17	6.242973	0	0	0	0	0	1	1	1
COL1A2	Collagen alpha-2(I) chain	129209.4	6.620338	1	0	1	0	0	0	1	0
ELA2	Leukocyte elastase	28499.79	9.414955	0	0	0	1	1	1	0	0
CD14	Monocyte differentiation antigen CD14	40050.74	5.555	0	0	1	1	0	0	1	0
KRT7	Keratin, type II cytoskeletal 7	51386.38	4.964653	0	0	0	1	0	1	0	1
FBP1	Fructose-1,6-bisphosphatase 1	36790.79	4.66291	0	1	0	1	0	1	0	0
SPARC	SPARC	34609.71	4.492226	0	0	1	1	0	0	1	0
C4A	Complement C4-A	192650.5	5.115257	0	0	0	1	0	1	0	1
H2AFZ	Histone H2A.Z	13544.55	10.51362	0	1	0	1	0	1	0	0
C9orf14	Putative uncharacterized protein C9orf14	12074.48	9.732613	0	1	0	1	0	1	0	0
IL8	Interleukin-8	11090.93	8.913689	0	0	1	1	0	0	1	0
TROVE2	60 kDa SS-A/Ro ribonucleoprotein	60631.2	5.970497	0	0	0	0	1	1	0	1
TXN	Thioredoxin	11729.74	4.686409	0	1	0	1	0	1	0	0

HSPD1	60 kDa heat shock protein, mitochondrial	61016.38	5.174858	1	1	0	0	0	1	0	0
PABPC1	Polyadenylate-binding protein 1	70625.87	7.163475	0	0	0	1	1	0	0	1
KRT3	Keratin, type II cytoskeletal 3	64464.59	4.772285	0	1	1	1	0	0	0	0
AKR1B1	Aldose reductase	35830.49	5.13192	1	0	0	0	1	0	0	1
TPSAB1	Tryptase alpha-1	30752.66	4.975121	0	1	1	0	0	0	0	1
TIMP2	Metalloproteinase inhibitor 2	24383.13	5.225914	0	1	1	1	0	0	0	0
CAPN2	Calpain-2 catalytic subunit	79958.81	4.506432	0	1	1	0	0	1	0	0
HK1	Hexokinase-1	102420.1	5.313179	0	1	0	1	0	1	0	0
PCMT1	Protein-L-isoaspartate(D-aspartate) O-methyltransferase	24634.59	5.386879	0	1	0	0	0	1	1	0
HNRNPA2B1	Heterogeneous nuclear ribonucleoproteins A2/B1	37406.73	4.740883	0	0	1	0	1	1	0	0
TGM1	Protein-glutamine gamma-glutamyltransferase K	89730.31	4.713646	1	0	1	0	0	0	1	0
CES1	Liver carboxylesterase 1	62481.15	5.107567	0	1	0	0	1	0	1	0
PSMA3	Proteasome subunit alpha type-3	28415.08	4.418312	0	0	0	1	1	1	0	0
PTBP1	Polypyrimidine tract-binding protein 1	57185.64	6.952522	0	0	0	0	1	1	1	0
TARS	Threonyl-tRNA synthetase, cytoplasmic	83381.86	5.069542	0	0	1	0	1	1	0	0
PSMB5	Proteasome subunit beta type-5	28462.21	4.215797	0	0	0	1	0	1	1	0
LAP3	Cytosol aminopeptidase	56130.81	5.640877	0	0	0	0	1	1	0	1
ERP29	Endoplasmic reticulum protein ERp29	28975.15	4.757439	0	1	0	0	1	1	0	0
CORO1A	Coronin-1A	50993.8	5.35836	0	1	0	1	0	1	0	0
MAT2A	S-adenosylmethionine synthetase isoform type-2	43633.33	4.901741	0	1	0	0	0	1	1	0
PGM1	Phosphoglucomutase-1	61410.53	4.926308	0	1	0	0	1	0	0	1
SERPINF1	Pigment epithelium-derived factor	46313.3	5.024147	0	0	1	0	1	0	1	0
COL15A1	Collagen alpha-1(XV) chain	141631.7	4.9	0	1	1	0	1	0	0	0
DCI	3,2-trans-enoyl-CoA isomerase, mitochondrial	32795.24	5.994637	0	1	0	1	0	1	0	0
PPIC	Peptidyl-prolyl cis-trans isomerase C	22748.84	6.188713	0	0	1	1	0	0	1	0

ARCN1	Coatomer subunit delta	57174.12	5.190025	0	0	1	0	0	1	0	1
GSS	Glutathione synthetase	52352.26	4.89875	0	1	0	0	0	1	1	0
FASN	Fatty acid synthase	273227.2	5.132881	0	1	0	1	0	1	0	0
PSMB3	Proteasome subunit beta type-3	22933.45	4.617409	0	0	1	1	0	1	0	0
SERPINB8	Serpin B8	42739.19	5.41	0	1	0	1	1	0	0	0
ANXA11	Annexin A11	54355.07	4.452171	0	1	1	1	0	0	0	0
STC1	Stanniocalcin-1	27603.78	6.384819	0	0	1	1	0	1	0	0
AKR1C2	Aldo-keto reductase family 1 member C2	36711.96	5.140038	0	0	1	0	0	0	1	1
DEFA1	Neutrophil defensin 1	10194.18	4.873436	0	0	0	1	1	0	1	0
UBE2N	Ubiquitin-conjugating enzyme E2 N	17126.97	5.0093	0	0	1	1	0	0	1	0
ARF3	ADP-rib ylation factor 3	20587.73	6.84	1	0	0	1	1	0	0	0
B2M	Beta-2-microglobulin	13705.91	4.750069	0	0	1	1	0	1	0	0
ACTG1	Actin, cytoplasmic 2	41765.79	5.31	1	0	1	0	1	0	0	0
TPM4	Tropomyosin alpha-4 chain	28504.49	4.585045	0	1	0	0	0	1	1	0
SPTBN1	Spectrin beta chain, brain 1	274438.7	4.995842	0	0	0	1	1	1	0	0
TAGLN	Transgelin	22596.43	5.685524	0	0	1	0	0	1	1	0
DSC2	Desmocollin-2	99898.74	4.552361	1	0	1	1	0	0	0	0
YWHAH	14-3-3 protein eta	28201.02	4.245598	0	0	1	0	0	1	0	1
BAX	Apoptosis regulator BAX	21170.76	4.811058	0	0	0	1	0	0	1	1
ARHGAP1	Rho GTPase-activating protein 1	50404.19	4.724968	0	1	1	0	0	1	0	0
ILF3	Interleukin enhancer-binding factor 3	95279.07	5.216621	0	0	0	0	1	1	0	1
ECH1	Delta(3,5)-Delta(2,4)-dienoyl-CoA isomerase, mitochondrial	35793.38	5.617165	0	1	0	0	0	1	1	0
UBE2V1	Ubiquitin-conjugating enzyme E2 variant 1	25780.04	5.822029	0	0	1	1	0	0	1	0
LAMB3	Laminin subunit beta-3	129488.6	5.495506	0	0	1	1	0	1	0	0
LAMC2	Laminin subunit gamma-2	130891.7	4.975441	0	0	1	0	0	1	1	0
TRIM29	Tripartite motif-containing protein 29	65793.3	5.332405	1	0	0	0	1	1	0	0
PTGR1	Prostaglandin reductase 1	35846.73	4.927803	1	0	0	0	0	1	0	1
PPP2R4	Serine/threonine-protein phosphatase 2A	40641.44	4.830391	0	0	0	0	1	1	0	1

	regulatory subunit B'										
SEPT7	Septin-7	50647.99	6.031166	0	0	0	1	0	1	0	1
VASN	Vasorin	71667.52	5.769157	0	1	1	1	0	0	0	0
GBP6	Guanylate-binding protein 6	72380.85	5.266823	1	0	1	1	0	0	0	0
KRT73	Keratin, type II cytoskeletal 73	58886.74	4.967323	0	1	0	1	0	0	0	1
HRNR	Hornerin	282227.6	7.005287	0	0	1	1	0	0	0	1
KRT78	Keratin, type II cytoskeletal 78	56830.49	5.035042	0	1	0	1	0	0	0	1
EVPL	Envoplakin	231477	5.581703	1	1	0	1	0	0	0	0
KLK6	Kallikrein-6	26838.36	5.783363	1	0	1	1	0	0	0	0
KCTD12	BTB/POZ domain-containing protein KCTD12	35678.69	4.496178	0	1	1	0	0	1	0	0
ABHD8	Abhydrolase domain-containing protein 8	47301.09	5.538979	0	1	0	1	0	1	0	0
ACO2	Aconitate hydratase, mitochondrial	85371.92	5.605629	0	0	0	1	0	1	0	1
TXNDC4	Thioredoxin domain-containing protein 4	46941.4	4.558342	0	0	0	0	1	1	1	0
ACAT2	Acetyl-CoA acetyltransferase, cytosolic	41324.39	5.946999	0	1	0	0	0	1	1	0
RNPEP	Aminopeptidase B	72549.46	4.588783	0	0	0	0	1	1	1	0
MUC5B	Mucin-5B	590122.1	5.249733	0	0	0	0	1	0	1	1
OLA1	Obg-like ATPase 1	44715.3	5.507683	0	0	0	1	0	0	1	1
MAT2B	Methionine adenosyltransferase 2 subunit beta	37528.18	6.007774	0	0	0	0	1	1	0	1
DMBT1	Deleted in malignant brain tumors 1 protein	260568.8	4.433479	0	0	0	1	0	1	1	0
GTF2IRD1	General transcription factor II-I repeat domain-containing protein 1	105990.7	5.309868	0	0	1	1	0	0	0	1
KLK13	Kallikrein-13	30550.51	6.182945	1	0	1	1	0	0	0	0
EIF3L	Eukaryotic translation initiation factor 3 subunit L	66684.41	4.114113	0	0	1	0	0	1	1	0
DRG1	Developmentally-regulated GTP-binding protein 1	40516.82	6.221931	0	0	0	0	0	1	1	1
DDTL	D-dopachrome decarboxylase-like protein	14186.27	5.555962	0	0	0	0	0	1	1	0
UNCX	Homeobox protein unc-4 homolog	53656.79	6.12249	0	0	1	0	0	1	0	0
TMPRSS11	Transmembrane protease, serine 11E2	47656.08	5.80291	0	0	1	1	0	0	0	0

E2

	Putative rhophilin-2-like protein	65902.53	4.982597	0	1	0	1	0	0	0	0
PSMD11	26S proteasome non-ATPase regulatory subunit 11	47434.04	4.889565	0	1	1	0	0	0	0	0
XPO1	Exportin-1	123306.1	4.92823	0	0	0	0	0	1	1	0
PFAS	Phosphoribosylformylglycinamidine synthase	144572.8	5.041023	0	0	0	0	1	1	0	0
TXNL1	Thioredoxin-like protein 1	32230.73	4.480797	0	1	0	0	0	1	0	0
H2AFY	Core histone macro-H2A.1	39592.47	9.636909	0	0	0	1	0	1	0	0
SH3BGRL	SH3 domain-binding glutamic acid-rich-like protein	12766.38	4.477592	0	1	0	0	0	1	0	0
LYPLA1	Acyl-protein thioesterase 1	24653.49	5.621437	0	0	0	0	0	1	1	0
	CRNN	Cornulin	53501.64	1	1	0	0	0	0	0	0
LYPD3	Ly6/PLAUR domain-containing protein 3	35947.62	5.862511	1	0	1	0	0	0	0	0
AHSA1	Activator of 90 kDa heat shock protein ATPase homolog 1	38250.25	4.948631	0	0	0	0	0	1	1	0
ASMTL	N-acetylserotonin O-methyltransferase-like protein	68812.95	4.958244	0	0	0	0	1	0	1	0
CFD	Complement factor D	27015.86	6.398064	1	0	1	0	0	0	0	0
INS	Insulin	11973.02	5.22	1	0	0	0	1	0	0	0
	Ig kappa chain V-I region AG	11984.88	4.028664	0	0	1	1	0	0	0	0
	Ig kappa chain V-III region NG9 (Fragment)	10722.28	6.3	1	0	0	0	1	0	0	0
	Ig kappa chain V-IV region Len	12632.19	3.508385	0	0	1	1	0	0	0	0
	Ig lambda chain V-I region HA	11888.89	4.911461	0	0	0	0	1	1	0	0
	Ig heavy chain V-III region TIL	12238.88	4.29644	0	0	1	0	0	0	1	0
IGHD	Ig delta chain C region	42227.24	5.96003	0	0	0	1	0	1	0	0
APOH	Beta-2-glycoprotein 1	38272.66	5.320549	0	0	1	1	0	0	0	0
ORM1	Alpha-1-acid glycoprotein 1	23496.76	4.228615	0	0	0	1	0	0	1	0
AHSG	Alpha-2-HS-glycoprotein	39299.71	4.908897	0	0	1	0	0	1	0	0
A1BG	Alpha-1B-glycoprotein	54238.58	5.013786	1	0	0	1	0	0	0	0
KRT6B	Keratin, type II cytoskeletal 6B	59989.26	5.030342	0	0	1	1	0	0	0	0

	Ig kappa chain V-III region VG (Fragment)	12567.3	4.85	1	0	0	0	1	0	0	0
	Ig kappa chain V-III region VH (Fragment)	12749.41	5.64	0	0	1	0	1	0	0	0
APP	Amyloid beta A4 protein	86888.05	4.458046	0	0	1	0	0	1	0	0
SLC25A5	ADP/ATP translocase 2	32874.17	8.818092	1	0	0	1	0	0	0	0
	Ig kappa chain V-II region RPMI 6410	14697.37	9.34	1	0	0	0	1	0	0	0
PYGL	Glycogen phosphorylase, liver form	97087.03	4.93795	0	1	0	0	0	1	0	0
TUBB	Tubulin beta chain	49638.97	4.282127	0	0	1	0	0	1	0	0
PRSS1	Trypsin-1	26541.09	6.08	1	0	0	0	1	0	0	0
PSAP	Proactivator polypeptide	58073.85	5.06	0	0	1	0	1	0	0	0
HEXB	Beta-hexosaminidase subunit beta	63071.18	4.729774	0	0	0	0	0	1	1	0
BPGM	Bisphosphoglycerate mutase	29986.51	5.172615	0	1	0	0	0	1	0	0
CTSB	Cathepsin B	37796.83	4.518501	0	0	1	1	0	0	0	0
CTSG	Cathepsin G	28819.07	11.19	0	0	0	1	1	0	0	0
INHBA	Inhibin beta A chain	47412.01	6.740288	0	0	1	0	0	1	0	0
CFH	Complement factor H	139004.7	4.542747	0	0	1	1	0	0	0	0
CXCL1	Growth-regulated alpha protein	11294.22	10.45957	0	0	1	1	0	0	0	0
TPM1	Tropomy in alpha-1 chain	32688.68	4.69	1	0	0	0	1	0	0	0
DLD	Dihydrolipoyl dehydrogenase, mitochondrial	54115.92	5.806648	0	0	0	0	0	1	0	1
C1S	Complement C1s subcomponent	76634.8	4.188988	0	1	1	0	0	0	0	0
PARP1	Poly [ADP-ribose] polymerase 1	113012.4	6.115334	0	0	0	1	0	0	0	1
CLU	Clusterin	52461.01	5.174324	0	0	0	1	0	1	0	0
PCNA	Proliferating cell nuclear antigen	28750.29	4.257668	0	0	0	0	1	1	0	0
CKB	Creatine kinase B-type	42617.32	4.840645	0	0	0	0	0	0	1	1
VCAN	Versican core protein	372589.5	4.182365	0	0	0	0	1	0	1	0
PRKCSH	Glucosidase 2 subunit beta	59387.82	4.096916	0	1	0	0	0	1	0	0
IDE	Insulin-degrading enzyme	117893.3	4.902596	0	0	0	0	1	1	0	0
CPA3	Mast cell carboxypeptidase A	48668.78	5.521355	0	0	1	1	0	0	0	0
RAC2	Ras-related C3 botulinum toxin substrate 2	21415.08	4.762032	0	0	1	1	0	0	0	0

ARSA	Arylsulfatase A	53553.68	4.642509	0	0	0	0	0	1	0	1
MUC1	Mucin-1	121999.1	6.325432	1	0	0	1	0	0	0	0
H2AFX	Histone H2A.x	15135.41	10.6262	0	1	0	0	0	0	1	0
ITGB4	Integrin beta-4	202022.6	4.905693	0	0	0	0	0	0	1	1
NCL	Nucleolin	76568.36	4.513695	0	0	0	0	0	1	1	0
ITIH2	Inter-alpha-trypsin inhibitor heavy chain H2	106369.6	5.126686	0	0	1	0	0	0	1	0
PI3	Elafin	12261.39	9.143974	0	0	1	0	0	0	1	0
ANXA7	Annexin A7	52705.75	3.905403	0	0	0	1	0	1	0	0
MX1	Interferon-induced GTP-binding protein Mx1	75487.23	5.022758	0	0	0	0	0	1	0	1
FUT3	Galactoside 3(4)-L-fucosyltransferase	42090.24	5.8592	0	0	1	1	0	0	0	0
CD9	CD9 antigen	25398.98	4.923958	0	0	1	1	0	0	0	0
FBLN1	Fibulin-1	77162.35	4.515617	0	0	0	0	1	0	1	0
AZGP1	Zinc-alpha-2-glycoprotein	33850.89	4.33564	0	1	0	0	0	1	0	0
PSMB9	Proteasome subunit beta type-9	23249.54	4.203087	0	0	0	0	1	1	0	0
PSMA5	Proteasome subunit alpha type-5	26394.2	4.517433	0	0	0	0	1	1	0	0
PSMB6	Proteasome subunit beta type-6	25341.45	4.135796	0	0	1	1	0	0	0	0
MAPK1	Mitogen-activated protein kinase 1	41363.22	4.818214	0	0	0	1	0	1	0	0
IMPA1	Inositol monophosphatase	30169.17	4.70916	0	0	0	0	1	1	0	0
EEF1D	Elongation factor 1-delta	31102.78	4.711617	0	0	0	0	1	1	0	0
ADSS	Adenylosuccinate synthetase isozyme 2	50065.77	4.864464	0	0	0	0	1	1	0	0
GSTT1	Glutathione S-transferase theta-1	27317.56	5.35334	0	0	1	0	0	1	0	0
GDI1	Rab GDP dissociation inhibitor alpha	50550.14	4.185677	0	0	0	0	0	1	1	0
CTNNA1	Catenin alpha-1	100008.5	5.224739	0	0	1	0	0	1	0	0
SPRR1A	Cornifin-A	9870.877	8.84565	0	0	1	1	0	0	0	0
THBS2	Thrombospondin-2	129907.8	4.275932	0	1	1	0	0	0	0	0
HSPA9	Stress-70 protein, mitochondrial	73634.77	5.185326	0	1	1	0	0	0	0	0
EIF2S3	Eukaryotic translation initiation factor 2 subunit 3	51077.22	6.612968	0	0	0	0	0	1	1	0
AKR1C3	Aldo-keto reductase family 1 member C3	36820.84	5.211494	0	0	0	0	0	1	0	1

PAFAH1B1	Platelet-activating factor acetylhydrolase IB subunit alpha	46608.16	5.82737	0	0	0	0	0	1	1	0
LGALS7	Galectin-7	15065.8	6.584129	0	1	1	0	0	0	0	0
SERPINB4	Serpin B4	44825.48	5.86	1	0	0	0	1	0	0	0
CCT5	T-complex protein 1 subunit epsilon	59632.81	5.14463	0	1	0	0	0	1	0	0
ALDH9A1	4-trimethylaminobutyraldehyde dehydrogenase	53766.98	4.803688	0	1	1	0	0	0	0	0
SERPINH1	Serpin H1	46411.18	6.174614	0	0	0	0	0	1	1	0
RAB7A	Ras-related protein Rab-7a	23474.84	4.831139	0	1	0	1	0	0	0	0
THOP1	Thimet oligopeptidase	78789.48	4.829109	0	0	0	0	0	1	0	1
ACLY	ATP-citrate synthase	120761.9	6.95	0	0	0	1	1	0	0	0
CTSC	Dipeptidyl-peptidase 1	51808.25	4.437431	0	0	1	0	0	1	0	0
EIF6	Eukaryotic translation initiation factor 6	26582.19	4.334679	0	0	0	0	0	1	1	0
MYL6	My in light polypeptide 6	16919.13	4.56	0	0	0	1	1	0	0	0
EIF4A1	Eukaryotic initiation factor 4A-I	46124.55	4.682991	0	0	1	0	0	1	0	0
S100A10	Protein S100-A10	11195.51	5.855354	1	0	0	0	0	1	0	0
DSTN	Destrin	18493.48	5.795433	0	0	1	0	0	0	1	0
RAB10	Ras-related protein Rab-10	22526.59	6.322121	0	1	0	1	0	0	0	0
SNRPD1	Small nuclear ribonucleoprotein Sm D1	13273.36	11.5608	0	0	0	0	0	0	1	1
RAB11A	Ras-related protein Rab-11A	24378.41	4.845772	1	0	0	0	0	1	0	0
UBE2L3	Ubiquitin-conjugating enzyme E2 L3	17850.24	5.784004	0	1	0	0	0	1	0	0
DCD	Dermcidin	11276.83	5.624962	0	0	1	1	0	0	0	0
H3F3A	Histone H3.3	15318.5	11.24709	0	0	0	0	1	0	1	0
HNRNPU	Heterogeneous nuclear ribonucleoprotein U	90456.97	4.900673	0	0	0	0	0	1	1	0
EIF4G1	Eukaryotic translation initiation factor 4 gamma 1	175426.4	4.899712	0	0	0	0	0	1	0	1
GLO1	Lactoylglutathione lyase	20764.25	4.562294	0	0	0	0	1	0	1	0
AHNAK	Neuroblast differentiation-associated protein AHNAK	628699.4	5.714577	1	0	0	1	0	0	0	0
DPYD	Dihydropyrimidine dehydrogenase [NADP+]	111329.3	5.06057	0	0	0	0	1	1	0	0

PRDX4	Peroxiredoxin-4	30520.81	4.860405	0	0	0	0	1	1	0	0
PSMD2	26S proteasome non-ATPase regulatory subunit 2	100135.8	4.600426	0	0	0	1	0	1	0	0
PKP1	Plakophilin-1	82807.83	6.571312	0	0	1	0	0	0	1	0
EIF4A2	Eukaryotic initiation factor 4A-II	46372.78	4.708092	0	0	0	0	1	1	0	0
NUMA1	Nuclear mitotic apparatus protein 1	238115.2	5.413902	0	0	0	0	1	1	0	0
PCOLCE	Procollagen C-endopeptidase enhancer 1	47941.98	4.997551	0	0	1	0	0	0	1	0
PCBP1	Poly(rC)-binding protein 1	37473.94	5.67762	0	0	1	1	0	0	0	0
IGFBP7	Insulin-like growth factor-binding protein 7	29111.44	6.121742	0	0	1	1	0	0	0	0
LAMA4	Laminin subunit alpha-4	202400.6	4.901955	0	0	1	1	0	0	0	0
PSMD5	26S proteasome non-ATPase regulatory subunit 5	56160.36	4.6894	0	0	1	0	0	0	0	1
FSCN1	Fascin	54496.05	5.135658	0	0	1	0	0	1	0	0
KYNU	Kynureninase	52318.18	5.376625	0	0	0	0	0	1	0	1
LAMA3	Laminin subunit alpha-3	366414	5.373314	0	0	0	0	0	1	1	0
HSP90AB2P	Putative heat shock protein HSP 90-beta 2	44321.11	4.430595	0	0	0	0	0	1	1	0
NAPRT1	Nicotinate phosphoribosyltransferase	57542.01	4.84214	0	0	1	0	0	0	1	0
CD109	CD109 antigen	161587.3	4.555992	0	0	1	0	0	1	0	0
MOBKL1A	Mps one binder kinase activator-like 1A	25074.64	5.09037	0	0	0	0	1	1	0	0
KRT74	Keratin, type II cytoskeletal 74	57829.64	5.314995	0	1	0	1	0	0	0	0
ABI3BP	Target of Nesh-SH3	118568.8	8.814781	0	0	1	1	0	0	0	0
SLURP2	Secreted Ly-6/uPAR-related protein 2	10152.84	5.845528	0	0	1	0	0	0	1	0
AEBP1	Adipocyte enhancer-binding protein 1	130847	4.61132	0	1	1	0	0	0	0	0
KATNAL2	Katanin p60 ATPase-containing subunit A-like 2	61214.5	7.22	1	0	0	0	1	0	0	0
BPIL1	Bactericidal/permeability-increasing protein-like 1	49141.72	6.251518	0	1	0	0	0	1	0	0
SCFD1	Sec1 family domain-containing protein 1	72334.05	4.748573	0	0	1	0	0	1	0	0
ANP32B	Acidic leucine-rich nuclear phosphoprotein 32	28770.31	3.870689	0	1	0	0	0	1	0	0

	family member B										
DCPS	Scavenger mRNA-decapping enzyme DcpS	38585.02	4.967751	0	0	0	0	0	1	0	1
HNRNPAB	Heterogeneous nuclear ribonucleoprotein A/B	36202.41	4.572762	0	0	0	0	1	1	0	0
CCT7	T-complex protein 1 subunit eta	59328.92	5.557564	0	0	0	1	0	0	0	1
	Uncharacterized protein MGC16385	13715.74	4.32	1	0	0	0	1	0	0	0
H2AFJ	Histone H2A.J	14010.93	10.9	1	0	0	0	1	0	0	0
NIF3L1	NIF3-like protein 1	41941.5	5.454811	0	0	0	0	0	1	0	1
HDHD2	Haloacid dehalogenase-like hydrolase domain-containing protein 2	28517.94	5.84	0	0	0	1	1	0	0	0
SDCBP2	Syntenin-2	31573.75	7.156319	1	0	0	1	0	0	0	0
SPON1	Spondin-1	90913.59	5.85	0	0	1	0	1	0	0	0
IL1F6	Interleukin-1 family member 6	17673.01	5.073921	0	1	1	0	0	0	0	0
ENOPH1	Enolase-phosphatase E1	28914.47	4.297935	0	0	1	0	0	1	0	0
AK3	GTP:AMP phosphotransferase mitochondrial	25549.55	5.601891	0	0	0	0	0	1	1	0
	ERP29	Endoplasmic reticulum protein ERp29	28975.15	1	1	0	0	0	0	0	0
VDAC3	Voltage-dependent anion-selective channel protein 3	30639.29	5.203377	1	0	0	1	0	0	0	0
DERA	Putative deoxyribose-phosphate aldolase	35208.47	6.714226	0	0	0	0	0	1	0	1
UFC1	Ufm1-conjugating enzyme 1	19446.02	5.231789	0	0	0	0	1	1	0	0
SCIN	Adseverin	80438.44	4.68235	0	1	0	1	0	0	0	0
ARHGAP10	Rho GTPase-activating protein 10	89318.34	5.622078	0	0	0	0	0	0	1	0
	Keratin-81-like protein	53375.16	5.085457	0	0	0	0	0	0	0	1
	NPIP-like protein ENSP00000346774	25989.15	11.58814	0	0	0	0	0	1	0	0
	Uncharacterized protein ENSP00000361571	66294.42	5.014534	0	0	1	0	0	0	0	0
	KRT4	Keratin, type II	57249.83	0	1	0	0	0	0	0	0

	ACTN4	cytoskeletal 4 Alpha-actinin-4	104788.5	0	1	0	0	0	0	0	0
SEPT13	Septin-13	33130.29	8.76	0	0	0	0	1	0	0	0
EML1	Echinoderm microtubule-associated protein-like 1	89762.76	5.350243	0	0	0	0	0	1	0	0
CACNA1A	Voltage-dependent P/Q-type calcium channel subunit alpha-1A	282185.2	5.902992	0	0	0	0	0	0	1	0
	ENO1	Alpha-enolase	47139.32	0	1	0	0	0	0	0	0
CES2	Carboxylesterase 2	61767.23	5.005241	0	0	1	0	0	0	0	0
PDXK	Pyridoxal kinase	35079.91	4.841499	0	0	0	0	0	1	0	0
COPE	Coatomer subunit epsilon	34460.34	4.399406	0	0	0	0	0	1	0	0
OPLAH	5-oxoprolinase	137370.6	5.285942	0	0	0	0	0	1	0	0
GIPC1	PDZ domain-containing protein GIPC1	36026.69	5.9	1	0	0	0	0	0	0	0
MYLC2B	Myosin regulatory light chain MRLC2	19766.52	4.472466	0	0	0	1	0	0	0	0
ARPC1B	Actin-related protein 2/3 complex subunit 1B	40923.41	7.542656	0	0	0	0	0	1	0	0
EIF3H	Eukaryotic translation initiation factor 3 subunit H	39905.05	4.869484	0	0	1	0	0	0	0	0
ARPC5	Actin-related protein 2/3 complex subunit 5	16310.3	4.889992	0	0	0	1	0	0	0	0
ZBTB24	Zinc finger and BTB domain-containing protein 24	78232.82	6.229088	0	0	0	0	0	0	0	1
TPD52L2	Tumor protein D54	22224.27	5.030769	0	0	0	0	0	1	0	0
CA12	Carbonic anhydrase 12	39426.05	5.152321	0	0	0	0	0	1	0	0
PLRG1	Pleiotropic regulator 1	57158.1	6.945366	0	0	0	0	0	1	0	0
NUDT21	Cleavage and polyadenylation specificity factor subunit 5	26210.74	5.23307	0	0	0	0	0	1	0	0
NRD1	Nardilysin	131488.5	4.89	0	0	0	0	1	0	0	0
KALRN	Kalirin	339960.3	5.057045	0	0	0	0	0	1	0	0
DIAPH1	Protein diaphanous homolog 1	141257.5	4.99894	0	0	0	1	0	0	0	0

UGDH	UDP-glucose 6-dehydrogenase	54989.25	5.077232	0	0	0	0	0	0	0	1
CTSL2	Cathepsin L2	37305.12	5.730492	0	0	0	0	0	1	0	0
	ACTN1	Alpha-actinin-1	102992.6	0	1	0	0	0	0	0	0
CRTAP	Cartilage-associated protein	46531.94	4.348778	0	0	0	1	0	0	0	0
ATP6AP2	Renin receptor	38983.32	4.603203	0	0	0	0	0	1	0	0
GLRX3	Glutaredoxin-3	37408.23	5.31	0	0	0	0	1	0	0	0
KRT33A	Keratin, type I cuticular Ha3-I	45911.39	4.437752	0	0	0	1	0	0	0	0
SEC14L2	SEC14-like protein 2	46115.56	5.054268	0	0	0	0	0	1	0	0
DDAH1	N(G),N(G)-dimethylarginine dimethylaminohydrolase 1	31101.93	5.002998	0	0	0	0	0	1	0	0
PRKD3	Serine/threonine-protein kinase D3	100406.2	6.42	1	0	0	0	0	0	0	0
LETM1	LETM1 and EF-hand domain-containing protein 1, mitochondrial	83301.96	6.3	1	0	0	0	0	0	0	0
UTS2	Urotensin-2	14286.44	7.67	0	0	0	0	1	0	0	0
ABCA1	ATP-binding cassette sub-family A member 1	254123	4.941154	0	0	0	1	0	0	0	0
KRT75	Keratin, type II cytoskeletal 75	59467.97	5.214058	0	0	0	1	0	0	0	0
AIFM1	Apoptosis-inducing factor 1, mitochondrial	66858.91	6.274696	0	0	0	0	0	1	0	0
DDAH2	N(G),N(G)-dimethylarginine dimethylaminohydrolase 2	29625.45	5.506721	0	0	0	0	0	1	0	0
ADH1C	Alcohol dehydrogenase 1C	39841.67	8.63	0	0	0	0	1	0	0	0
MT-CO2	Cytochrome c oxidase subunit 2	25548.21	4.67	1	0	0	0	0	0	0	0
EGFR	Epidermal growth factor receptor	134190.3	6.26	0	0	0	0	1	0	0	0
F2	Prothrombin	69992.12	4.791832	0	0	0	0	0	1	0	0
C1R	Complement C1r subcomponent	80066.81	4.481331	0	0	1	0	0	0	0	0
ADA	Adenosine deaminase	40738.66	4.665794	0	0	0	0	0	1	0	0
CST3	Cystatin-C	15789.08	6.808968	0	0	1	0	0	0	0	0
LDLR	Low-density lipoprotein receptor	95313.63	4.544563	0	0	0	0	0	1	0	0
	Ig lambda chain V-I region NIG-64	11446.54	4.66	0	0	0	0	1	0	0	0
	Ig lambda chain V-III region SH	11385.59	4.367256	0	0	0	0	0	1	0	0

	Ig heavy chain V-III region POM	12916.23	8.05	0	0	0	0	1	0	0	0
	Ig heavy chain V-III region GAL	12628.06	4.563683	0	0	0	0	0	0	0	1
HLA-B	HLA class I histocompatibility antigen, B-7 alpha chain	40434.95	4.589745	0	0	0	0	0	0	1	0
COL1A1	Collagen alpha-1(I) chain	138826.6	5.127968	0	0	0	0	0	0	1	0
APOA1	Apolipoprotein A-I	30758.93	5.56	1	0	0	0	0	0	0	0
SLC4A1	Band 3 anion transport protein	101727.4	4.569237	0	0	0	1	0	0	0	0
APCS	Serum amyloid P-component	25371.13	4.415535	0	0	1	0	0	0	0	0
C1QC	Complement C1q subcomponent subunit C	25757.14	8.61	1	0	0	0	0	0	0	0
AMBP	Protein AMBP	38973.98	5.95	1	0	0	0	0	0	0	0
TFRC	Transferrin receptor protein 1	84817.94	4.771751	0	0	0	0	0	0	1	0
HPX	Hemopexin	51643.27	6.55	1	0	0	0	0	0	0	0
C4BPA	C4b-binding protein alpha chain	66989.4	5.069221	0	0	1	0	0	0	0	0
SEMG1	Semenogelin-1	52099.88	6.897835	0	0	0	0	0	0	1	0
	P4HB	Protein disulfide-isomerase	57080.67	0	1	0	0	0	0	0	0
HLA-A	HLA class I histocompatibility antigen, A-3 alpha chain	40815.17	4.667183	0	0	0	0	0	1	0	0
CAPNS1	Calpain small subunit 1	28297.73	4.497887	0	0	0	0	0	1	0	0
AMY1A	Alpha-amylase 1	57730.91	4.745049	0	0	1	0	0	0	0	0
APOD	Apolipoprotein D	21261.76	5.06	1	0	0	0	0	0	0	0
	LDHB	L-lactate dehydrogenase B chain	36615.14	0	1	0	0	0	0	0	0
EIF2S1	Eukaryotic translation initiation factor 2 subunit 1	36089.37	4.558235	0	0	0	0	0	1	0	0
RPLP1	60S acidic rib omal protein P1	11506.69	4.26	0	0	0	0	1	0	0	0

SSB	Lupus La protein	46808.16	5.715004	0	0	0	0	0	1	0	0
KRT18	Keratin, type I cytoskeletal 18	48028.54	4.819923	0	0	0	0	0	1	0	0
UROD	Uroporphyrinogen decarboxylase	40760.83	5.77	0	0	0	0	1	0	0	0
IGKV4-1	Ig kappa chain V-IV region (Fragment)	13371.59	5.09	0	0	0	0	1	0	0	0
NPM1	Nucleophosmin	32554.84	4.513481	0	0	0	0	0	1	0	0
	YWHAE	14-3-3 protein epsilon	29155.42	0	1	0	0	0	0	0	0
C8G	Complement component C8 gamma chain	22263.54	5.679329	0	0	0	1	0	0	0	0
GUSB	Beta-glucuronidase	74684.69	6.54	0	0	0	0	1	0	0	0
	PEBP1	Phosphatidylethanolamine-binding protein 1	21043.67	0	1	0	0	0	0	0	0
HMBS	Porphobilinogen deaminase	39305.61	6.233147	0	0	0	0	0	1	0	0
GNAI3	Guanine nucleotide-binding protein G(k) subunit alpha	40506.27	4.630013	0	0	0	1	0	0	0	0
ENO2	Gamma-enolase	47239	4.464027	0	0	0	0	0	1	0	0
MMP7	Matrilysin	29657.87	5.168129	0	0	0	0	0	1	0	0
MMP10	Stromelysin-2	54116.79	4.556206	0	0	0	0	0	1	0	0
LGALS1	Galectin-1	14706.2	4.915306	0	0	1	0	0	0	0	0
RBP1	Retinol-binding protein 1	15839.9	4.685661	0	0	0	0	0	0	0	1
TACSTD2	Tumor-associated calcium signal transducer 2	35686.62	8.246758	0	0	0	0	0	1	0	0
CSF3	Granulocyte colony-stimulating factor	22278.51	5.043587	0	0	1	0	0	0	0	0
HNRPA1L3	Putative heterogeneous nuclear ribonucleoprotein A1-like protein 3	34202.28	9.23	0	0	0	0	1	0	0	0
GAA	Lysosomal alpha-glucosidase	105270.8	4.652229	0	0	0	0	0	1	0	0
HLA-B	HLA class I histocompatibility antigen, B-58 alpha chain	40312.04	4.712685	0	0	0	0	0	1	0	0

ESD	S-formylglutathione hydrolase	31442.46	4.699333	0	0	0	0	0	1	0	0
PYGB	Glycogen phosphorylase, brain form	96634.52	5.000755	0	0	0	0	0	1	0	0
G6PD	Glucose-6-phosphate 1-dehydrogenase	59218.99	4.922142	0	0	0	0	0	1	0	0
HARS	Histidyl-tRNA synthetase, cytoplasmic	57374.22	4.819817	0	0	0	0	0	1	0	0
COL6A2	Collagen alpha-2(VI) chain	108511.9	5.85	0	0	0	0	1	0	0	0
PIP	Prolactin-inducible protein	16561.8	4.749107	0	0	0	0	0	1	0	0
CEACAM1	Carcinoembryonic antigen-related cell adhesion molecule 1	57524.71	4.461143	0	0	0	0	0	0	1	0
TPT1	Translationally-controlled tumor protein	19582.58	4.329231	0	0	0	0	0	1	0	0
ETFA	Electron transfer flavoprotein subunit alpha, mitochondrial	35057.58	8.62	1	0	0	0	0	0	0	0
FDPS	Farnesyl pyroph phate synthetase	48244.63	5.83	0	0	0	0	1	0	0	0
SI	Sucrase-isomaltase, intestinal	209271	4.259483	0	0	0	0	0	1	0	0
NID1	Nidogen-1	136366.8	4.441277	0	0	0	1	0	0	0	0
	EIF5A	Eukaryotic translation initiation factor 5A-1	16821.4	0	1	0	0	0	0	0	0
ANPEP	Aminopeptidase N	109470.9	4.395988	0	0	0	0	0	1	0	0
	PPIA	Peptidyl-prolyl cis-trans isomerase A	18000.88	0	1	0	0	0	0	0	0
UCHL3	Ubiquitin carboxyl-terminal hydrolase isozyme L3	26165.97	4.542641	0	0	0	0	0	1	0	0
CHRNB2	Neuronal acetylcholine receptor subunit beta-2	56982.07	6.33	0	0	0	0	1	0	0	0

IGFBP3	Insulin-like growth factor-binding protein 3	31653.74	8.256371	0	0	0	0	0	1	0	0
CCT1	T-complex protein 1 subunit alpha	60305.58	5.151573	0	0	0	0	0	0	0	1
ITIH1	Inter-alpha-trypsin inhibitor heavy chain H1	101325.6	6.31	0	0	0	0	1	0	0	0
TPSB2	Tryptase beta-2	30495.47	5.157661	0	0	0	1	0	0	0	0
LMNB1	Lamin-B1	66367.62	4.773567	0	0	0	0	0	1	0	0
PTMS	Parathymosin	11523.19	4.13868	0	0	0	0	0	1	0	0
	Placental protein 11	42093.97	4.295158	0	0	1	0	0	0	0	0
ACO1	Cytoplasmic aconitate hydratase	98336.58	4.797386	0	0	1	0	0	0	0	0
PAICS	Multifunctional protein ADE2	47049.14	5.494972	0	0	0	0	0	0	1	0
UQCRC2	Cytochrome b-c1 complex subunit 2, mitochondrial	48412.88	8.74	1	0	0	0	0	0	0	0
MRC1	Macrophage mann e receptor 1	165904.8	6.11	0	0	0	0	1	0	0	0
ITGA6	Integrin alpha-6	126553.2	4.799736	0	0	0	0	0	1	0	0
RPS3	40S rib omal protein S3	26671.43	9.68	1	0	0	0	0	0	0	0
PRTN3	Myeloblastin	27789.27	7.688026	0	0	0	1	0	0	0	0
EEF1B2	Elongation factor 1-beta	24748.26	4.132271	0	0	0	0	0	1	0	0
	SOD3	Extracellular superoxide dismutase [Cu-Zn]	25834.67	0	1	0	0	0	0	0	0
CTSS	Cathepsin S	37471.3	5.214058	0	0	0	0	0	1	0	0
S100P	Protein S100-P	10393.22	4.491798	0	0	0	1	0	0	0	0
U2AF2	Splicing factor U2AF 65 kDa subunit	53467.19	6.535637	0	0	0	0	0	1	0	0
GSTM2	Glutathione S-transferase Mu 2	25727.98	4.529076	0	0	0	0	0	1	0	0
CRABP2	Cellular retinoic acid-binding protein 2	15683.02	5.074883	0	0	1	0	0	0	0	0
	ERO1L	ERO1-like	54358.05	0	1	0	0	0	0	0	0

		protein alpha									
C21orf33	ES1 protein homolog, mitochondrial	28152.73	7.19445	0	0	0	0	0	1	0	0
PPP2R1A	Serine/threonine-protein phosphatase 2A 65 kDa regulatory subunit A alpha isoform	65266.91	4.700401	0	0	1	0	0	0	0	0
ADSL	Adenylosuccinate lyase	54853.97	5.609047	0	0	0	0	0	1	0	0
GBP1	Interferon-induced guanylate-binding protein 1	67859.68	5.057899	0	0	0	0	0	0	1	0
GBP2	Interferon-induced guanylate-binding protein 2	67140.38	5.54	0	0	0	0	1	0	0	0
MAN1A1	Mannosyl-oligosaccharide 1,2-alpha-mannosidase IA	72922.28	5.059608	0	0	1	0	0	0	0	0
GPC1	Glypican-1	61611.01	5.472008	0	0	1	0	0	0	0	0
CTNNB1	Catenin beta-1	85442.21	4.920219	0	0	0	0	0	1	0	0
PHB	Prohibitin	29785.9	5.57	1	0	0	0	0	0	0	0
SERPINB6	Serpin B6	42594.06	4.768761	0	0	0	0	0	1	0	0
FBN1	Fibrillin-1	312097.5	4.81	0	0	0	0	1	0	0	0
COPB2	Coatomer subunit beta'	102422.5	4.557701	0	0	0	0	0	1	0	0
ACTN2	Alpha-actinin-2	103788.3	5.31	1	0	0	0	0	0	0	0
KRT20	Keratin, type I cytoskeletal 20	48456.92	4.873863	0	0	0	1	0	0	0	0
	IL1F6	Interleukin-1 family member 6	17673.01	0	1	0	0	0	0	0	0
CPOX	Coproporphyrinogen III oxidase, mitochondrial	50119.97	6.544182	0	0	0	0	0	0	0	1
PPP1CC	Serine/threonine-protein phosphatase PP1-gamma catalytic subunit	36959.72	4.835625	0	0	0	0	0	0	0	1
SRP14	Signal recognition particle 14 kDa protein	14560.82	9.927116	0	0	1	0	0	0	0	0
ATP6V1A	V-type proton ATPase catalytic subunit A	68260.49	4.510597	0	0	0	0	0	1	0	0

	LYZ	Lysozyme C	16526.28	0	1	0	0	0	0	0	0
MMP12	Macrophage metalloelastase	53967.14	5.133842	0	0	0	0	0	0	1	0
PSMB10	Proteasome subunit beta type-10	28918.07	5.986626	0	0	0	0	0	0	1	0
PRCP	Lys omal Pro-X carboxypeptidase	55763.92	6.76	0	0	0	0	1	0	0	0
CXCL5	C-X-C motif chemokine 5	11964.51	9.167473	0	0	0	1	0	0	0	0
MCAM	Cell surface glycoprotein MUC18	71562.6	5.58	0	0	0	0	1	0	0	0
VDAC2	Voltage-dependent anion-selective channel protein 2	31546.55	7.5	1	0	0	0	0	0	0	0
PSMD8	26S proteasome non-ATPase regulatory subunit 8	29985.62	6.85	1	0	0	0	0	0	0	0
	PITPNA	Phosphatidylinositol transfer protein alpha isoform	31786.02	0	1	0	0	0	0	0	0
IDH2	Isocitrate dehydrogenase [NADP], mitochondrial	50876.86	8.88	1	0	0	0	0	0	0	0
PITPNB	Phosphatidylinositol transfer protein beta isoform	31519.91	5.106178	0	0	0	0	0	1	0	0
	ABHD8	Abhydrolase domain-containing protein 8	47301.09	0	1	0	0	0	0	0	0
	RSU1	Ras suppressor protein 1	31520.69	0	1	0	0	0	0	0	0
AARS	Alanyl-tRNA synthetase, cytoplasmic	106743.2	4.737999	0	0	0	0	0	1	0	0

CARS	Cysteinyl-tRNA synthetase, cytoplasmic	85419.23	5.182976	0	0	0	0	0	1	0	0
	UGP2	UTP--glucose-1-phosphate uridylyltransferase	56904.72	0	1	0	0	0	0	0	0
HINT1	Histidine triad nucleotide-binding protein 1	13793.09	6.017174	0	0	0	0	0	1	0	0
NUP153	Nuclear pore complex protein Nup153	153843.2	8.97	1	0	0	0	0	0	0	0
KLK7	Kallikrein-7	27506.81	8.249855	0	0	1	0	0	0	0	0
CAMP	Cathelicidin antimicrobial peptide	19289.16	9.273109	0	0	1	0	0	0	0	0
		Uncharacterized protein FLJ39653	29535.18	0	1	0	0	0	0	0	0
CCT4	T-complex protein 1 subunit delta	57887.76	6.01226	0	0	1	0	0	0	0	0
DUSP3	Dual specificity protein phosphatase 3	20465.31	4.938591	0	0	1	0	0	0	0	0
HSD17B4	Peroxisomal multifunctional enzyme type 2	79636.3	5.980751	0	0	0	0	0	1	0	0
HNRNPF	Heterogeneous nuclear ribonucleoprotein F	45642.85	4.415855	0	0	0	0	0	1	0	0
ZNF132	Zinc finger protein 132	80570.98	8.77	0	0	0	0	1	0	0	0
SMS	Spermine synthase	41241.78	4.194221	0	0	0	0	0	1	0	0
PPP5C	Serine/threonine-protein phosphatase 5	56842.25	4.598717	0	0	0	0	0	1	0	0
RABGGTB	Geranylgeranyl transferase type-2 subunit beta	36900.31	4.371849	0	0	0	0	0	1	0	0
COPA	Coatomer subunit alpha	138257.9	7.7	1	0	0	0	0	0	0	0
SEC24C	Protein transport protein Sec24C	118239.1	4.578636	0	0	0	0	0	1	0	0
	ALDH2	Aldehyde dehydrogenase,	56345.62	0	1	0	0	0	0	0	0

		mitochon drial									
HSPA2	Heat shock-related 70 kDa protein 2	69977.86	4.87632	0	0	1	0	0	0	0	0
NAPA	Alpha-soluble NSF attachment protein	33211.28	4.48037	0	0	1	0	0	0	0	0
PLTP	Phospholipid transfer protein	54704.54	5.164818	0	0	0	0	0	1	0	0
SEC13	Protein SEC13 homolog	35518.11	5.22	0	0	0	0	1	0	0	0
EPPK1	Epiplakin	552753.7	4.858589	0	0	0	0	0	0	1	0
MTPN	Myotrophin	12886.61	4.791084	0	0	0	1	0	0	0	0
HIST1H2B D	Histone H2B type 1-D	13927.57	10.31	0	0	0	0	1	0	0	0
UBE2D3	Ubiquitin-conjugating enzyme E2 D3	16676.37	4.915413	0	0	0	0	0	0	1	0
HSPE1	10 kDa heat shock protein, mitochondrial	10924.87	5.374702	0	0	0	0	0	1	0	0
HNRNPK	Heterogeneous nuclear ribonucleoprotein K	50944.4	4.595406	0	0	0	0	0	1	0	0
PSMC6	26S protease regulatory subunit S10B	44145.1	7.09	1	0	0	0	0	0	0	0
RPL7A	60S rib omal protein L7a	29977.02	10.61	1	0	0	0	0	0	0	0
LCN6	Epididymal-specific lipocalin-6	18033.26	4.84	0	0	0	0	1	0	0	0
ESRRG	Estrogen-related receptor gamma	51272.63	4.85891	0	0	0	1	0	0	0	0
PPP2CB	Serine/threonine-protein phosphatase 2A catalytic subunit beta isoform	35552.33	4.343544	0	0	0	0	0	1	0	0
RAN	GTP-binding nuclear protein Ran	24407.62	5.193977	0	0	0	0	0	1	0	0
RPL30	60S ribosomal protein L30	12775.74	8.806984	0	0	1	0	0	0	0	0
RPL11	60S ribosomal protein L11	20239.64	9.095161	0	0	0	1	0	0	0	0
GRB2	Growth factor receptor-bound protein 2	25190.43	4.940514	0	0	0	0	0	1	0	0
UBE2I	SUMO-conjugating enzyme UBC9	17995.2	6.124199	0	0	0	1	0	0	0	0
TUBA4A	Tubulin alpha-4A chain	49892.37	4.338951	0	0	0	1	0	0	0	0
PAFAH1B2	Platelet-activating factor acetylhydrolase IB subunit beta	25553.12	5.57	0	0	0	0	1	0	0	0
PSPH	Ph ph erine ph phatase	24991.9	5.53	0	0	0	0	1	0	0	0
KRT83	Keratin, type II cuticular Hb3	54160.51	4.868629	0	0	0	1	0	0	0	0
RELN	Reelin	388166.5	4.550545	0	0	0	1	0	0	0	0

PRKDC	DNA-dependent protein kinase catalytic subunit	468787.9	6.75	1	0	0	0	0	0	0	0
LRP2	Low-density lipoprotein receptor-related protein 2	521615.6	4.89	1	0	0	0	0	0	0	0
SORD	Sorbitol dehydrogenase	38271.84	6.0756	0	0	0	0	0	1	0	0
SET	Protein SET	33468.7	4.079399	0	0	0	0	0	1	0	0
FKBP4	FK506-binding protein 4	51772.07	4.762566	0	0	0	0	0	1	0	0
MUC2	Mucin-2	539958.3	4.579277	0	0	0	0	0	0	1	0
NUCB1	Nucleobindin-1	53846.33	4.922142	0	0	1	0	0	0	0	0
ACY1	Aminoacylase-1	45855.99	5.77	0	0	0	0	1	0	0	0
LMNB2	Lamin-B2	67647.49	4.970421	0	0	0	0	0	1	0	0
	M6PRBP1	Mannose-6-phosphate receptor-binding protein 1	47017.91	0	1	0	0	0	0	0	0
GBE1	1,4-alpha-glucan-branching enzyme	80408.62	4.467552	0	0	0	0	0	1	0	0
AKR1C1	Aldo-keto reductase family 1 member C1	36765.02	5.160545	0	0	0	0	0	1	0	0
LRP1	Prolow-density lipoprotein receptor-related protein 1	504244.8	4.599037	0	0	0	0	0	1	0	0
AP1B1	AP-1 complex subunit beta-1	104540.1	4.455162	0	0	0	0	0	1	0	0
TWF1	Twinfilin-1	42182.55	5.043373	0	0	0	0	0	1	0	0
EFEMP1	EGF-containing fibulin-like extracellular matrix protein 1	54604.23	4.15139	0	0	0	0	0	1	0	0
BPTF	Nucleosome-remodeling factor subunit BPTF	338053.6	5.329308	0	0	0	0	0	1	0	0
LMAN2	Vesicular integral-membrane protein VIP36	40203.1	5.32621	0	0	0	1	0	0	0	0
EPS8	Epidermal growth factor receptor kinase substrate 8	91824.42	5.564186	0	0	0	0	0	0	1	0
GOLGA4	Golgin subfamily A member 4	260979.7	5.102974	0	0	0	1	0	0	0	0

COTL1	Coactosin-like protein	15935.03	4.850365	0	0	0	0	0	0	1	0
LY6D	Lymphocyte antigen 6D	13277.71	8.380806	0	0	0	0	0	0	1	0
SPARCL1	SPARC-like protein 1	75169.2	4.510704	0	0	1	0	0	0	0	0
MESDC2	Mesoderm development candidate 2	26060.3	5.50074	0	0	0	0	0	1	0	0
KRT72	Keratin, type II cytoskeletal 72	55842.44	5.077766	0	0	0	1	0	0	0	0
PSMD6	26S proteasome non-ATPase regulatory subunit 6	45502.27	4.482719	0	0	0	1	0	0	0	0
	SDC4	Syndecan -4	21628.03	0	1	0	0	0	0	0	0
PPA1	Inorganic pyrophosphatase	32639.15	4.746864	0	0	0	0	0	1	0	0
PPP1R7	Protein phosphatase 1 regulatory subunit 7	41538.53	4.680107	0	0	0	0	0	1	0	0
SEC23A	Protein transport protein Sec23A	86105.25	4.985588	0	0	0	0	0	1	0	0
SURF1	Surfeit locus protein 1	33310.39	9.64	0	0	0	0	1	0	0	0
GRK1	Rhodopsin kinase	63485.11	5.63	1	0	0	0	0	0	0	0
SYPL1	Synaptophysin-like protein 1	28546.7	8.69	1	0	0	0	0	0	0	0
PCK2	Phosphoenolpyruvate carboxykinase [GTP], mitochondrial	70591.61	5.966652	0	0	0	0	0	1	0	0
TXNRD1	Thioredoxin reductase 1, cytoplasmic	70824.35	5.051277	0	0	0	0	0	1	0	0
IMMT	Mitochondrial inner membrane protein	83626.35	6.08	1	0	0	0	0	0	0	0
CSPP1	Centr ome and spindle pole-associated protein 1	141663.6	6.26	0	0	0	0	1	0	0	0
TP53I3	Putative quinone oxidoreductase	35513.58	5.059181	0	0	0	0	0	0	0	1
ACTBL2	Beta-actin-like protein 2	41975.97	5.39	0	0	0	0	1	0	0	0
HSP90AA4P	Putative heat shock protein HSP 90-alpha A4	47682.12	4.628304	0	0	0	0	0	1	0	0
GNAS	Guanine nucleotide-binding protein G(s) subunit alpha isoforms XLas	110955.5	4.91	1	0	0	0	0	0	0	0
SNAP47	Synaptosomal-associated protein 47	47268.46	6.081047	0	0	0	0	0	1	0	0
MUC21	Mucin-21	54168.4	4.8	1	0	0	0	0	0	0	0
C10orf81	PH domain-containing protein C10orf81	51806.53	5.55	0	0	0	0	1	0	0	0

C9orf104	Uncharacterized protein C9orf104	6583.58	10.35	1	0	0	0	0	0	0	0
ANXA8L1	Annexin A8-like protein 1	36857.61	5.45	0	0	0	0	1	0	0	0
CROCC	Rootletin	228387.6	5.45	1	0	0	0	0	0	0	0
BROX	BRO1 domain-containing protein BROX	46446.65	4.9748	0	0	0	0	0	1	0	0
C1QL3	Complement C1q-like protein 3	26702.18	4.416283	0	0	1	0	0	0	0	0
KRT40	Keratin, type I cytoskeletal 40	48107.6	4.126289	0	0	1	0	0	0	0	0
CYB5R2	NADH-cytochrome b5 reductase 2	31438.37	5.450111	0	0	0	0	0	1	0	0
TWF2	Twinfilin-2	39523.27	5.257957	0	0	0	1	0	0	0	0
HIBCH	3-hydroxyisobutyryl-CoA hydrolase, mitochondrial	43454.4	6.155388	0	0	0	0	0	1	0	0
TATDN1	Putative deoxyribonuclease TATDN1	33579.93	5.622292	0	0	0	0	0	0	1	0
FAHD1	Fumarylacetoacetate hydrolase domain-containing protein 1	24826.73	5.163216	0	0	0	0	0	1	0	0
ATAD2	ATPase family AAA domain-containing protein 2	158456.1	5.146446	0	0	0	0	0	1	0	0
MICALCL	MICAL C-terminal-like protein	77520.97	8.64	0	0	0	0	1	0	0	0
	Uncharacterized protein LOC339524	28676.08	11.70297	0	0	0	0	0	0	0	1
HIST2H3A	Histone H3.2	15378.51	11.24709	0	0	0	0	0	0	1	0
SSH2	Protein phosphatase Slingshot homolog 2	158116.5	4.9011	0	0	0	1	0	0	0	0
GDPD3	Glycerophosphodiester phosphodiesterase domain-containing protein 3	29605.11	5.300362	0	0	0	1	0	0	0	0
GSTA5	Glutathione S-transferase A5	25705.6	7.74	1	0	0	0	0	0	0	0
	DCI	3,2-trans-enoyl-CoA isomerase, mitochondrial	32795.24	0	1	0	0	0	0	0	0
GALNT5	Polypeptide N-acetylgalactosaminyltransferase 5	106199.4	9.150383	0	0	0	0	0	1	0	0

RALYL	RNA-binding Raly-like protein	32310.56	7.69	1	0	0	0	0	0	0	0
ALPK2	Alpha-protein kinase 2	210038.6	4.820671	0	0	0	1	0	0	0	0
KTN1	Kinectin	156179.3	5.272377	0	0	0	1	0	0	0	0
CD163	Scavenger receptor cysteine-rich type 1 protein M130	125355.2	5.35537	0	0	1	0	0	0	0	0
DHX40	Probable ATP-dependent RNA helicase DHX40	88503.75	7.261101	0	0	0	0	0	1	0	0
FAM186B	Protein FAM186B	103656.6	6.78376	0	0	0	0	0	1	0	0
TMEFF1	Tomoregulin-1	40907.08	4.946709	0	0	0	0	0	0	1	0
ALDH16A1	Aldehyde dehydrogenase family 16 member A1	85059.68	5.455452	0	0	0	0	0	1	0	0
DOCK4	Dedicator of cytokinesis protein 4	225004.9	7.69	0	0	0	0	1	0	0	0
	TUFM	Elongation factor Tu, mitochondrial	49510.18	0	1	0	0	0	0	0	0
WDR31	WD repeat-containing protein 31	40813.42	8.65	0	0	0	0	1	0	0	0
C17orf76	Leucine-rich repeat-containing protein C17orf76	37756.66	6.37542	0	0	0	0	0	1	0	0
APOA1BP	Apolipoprotein A-I-binding protein	31654.24	4.661308	0	0	0	0	0	1	0	0
BVES	Blood vessel epicardial substance	41424.13	5.672493	0	0	0	1	0	0	0	0
MFSD8	Major facilitator superfamily domain-containing protein 8	57590.17	6.41	0	0	0	0	1	0	0	0
ACTL7C	Actin-like protein 7C	45538.47	5.221321	0	0	0	0	0	0	1	0
NT5C	5'(3')-deoxyribonucleotidase, cytosolic type	23368.07	5.532249	0	0	0	0	0	1	0	0
BICD2	Protein bicaudal D homolog 2	93476.03	4.927696	0	0	0	1	0	0	0	0
DSCAML1	Down syndrome cell adhesion molecule-like protein 1	224322.8	5.312325	0	0	0	0	0	0	1	0
ABCA5	ATP-binding cassette sub-family A member 5	186386.8	4.797493	0	0	0	0	0	0	0	1
TTN	Titin	3813810	6.01	1	0	0	0	0	0	0	0

DDB2	DNA damage-binding protein 2	47833.38	9.095695	0	0	0	0	0	1	0	0
FAM3C	Protein FAM3C	24664.58	5.769478	0	0	0	0	0	1	0	0
NDRG1	Protein NDRG1	42807.72	4.820457	0	0	0	0	0	1	0	0
PXDN	Peroxidasin homolog	165169.9	5.495079	0	0	0	0	0	1	0	0
KRT35	Keratin, type I cuticular Ha5	50323.49	4.483147	0	0	0	1	0	0	0	0
GOLGA1	Golgin subfamily A member 1	88146.15	5.11483	0	0	0	0	0	1	0	0
UFD1L	Ubiquitin fusion degradation protein 1 homolog	34478.31	5.039314	0	0	0	0	0	1	0	0
USP7	Ubiquitin carboxyl-terminal hydrolase 7	128220.3	4.682884	0	0	0	0	0	0	1	0
C19orf10	UPF0556 protein C19orf10	18783.31	5.00428	0	0	0	0	0	1	0	0
ISOC2	Isochorismatase domain-containing protein 2, mitochondrial	22322.84	6.413658	0	0	0	0	0	1	0	0
TMIGD2	Transmembrane and immunoglobulin domain-containing protein 2	30655.43	9.13	1	0	0	0	0	0	0	0
FAM3D	Protein FAM3D	24946.77	9.42	1	0	0	0	0	0	0	0
EFHD2	EF-hand domain-containing protein D2	26680.46	5.15	0	0	0	0	1	0	0	0
GALM	Aldose 1-epimerase	37742.13	5.015388	0	0	0	0	0	1	0	0
INTS12	Integrator complex subunit 12	48777.13	9.651543	0	0	0	0	0	0	0	1
S100A16	Protein S100-A16	11793.97	6.28	1	0	0	0	0	0	0	0
OTUB1	Ubiquitin thioesterase OTUB1	31264.42	4.216972	0	0	0	0	0	1	0	0
DCUN1D1	DCN1-like protein 1	30105.05	4.716743	0	0	0	0	0	1	0	0
AKR1CL2	Aldo-keto reductase family 1 member C-like protein 2	36551.65	6.89	0	0	0	0	1	0	0	0
TEKT5	Tektin-5	56258.24	5.539619	0	0	0	1	0	0	0	0
PVRL4	Poliovirus receptor-related protein 4	55419.78	4.819817	0	0	1	0	0	0	0	0
IPO9	Importin-9	115888.7	4.397484	0	0	0	0	0	1	0	0
KRT12	Keratin, type I cytoskeletal 12	53478.44	4.394814	0	0	0	1	0	0	0	0
CORO1B	Coronin-1B	54200.25	4.973305	0	0	0	0	0	1	0	0
TXNDC17	Thioredoxin domain-containing protein 17	13931.8	4.6847	0	0	1	0	0	0	0	0
CSTP1	Uncharacterized metallophosphoesterase	35553.88	4.994667	0	0	0	0	0	1	0	0

	CSTP1										
LXN	Latexin	25733.89	4.622749	0	0	0	0	0	1	0	0
COPS4	COP9 signalosome complex subunit 4	46239.67	4.697731	0	0	0	0	0	1	0	0
TMEM109	Transmembrane protein 109	26193.58	10.48	1	0	0	0	0	0	0	0
CARD11	Caspase recruitment domain-containing protein 11	133200.3	5.039207	0	0	0	0	0	1	0	0
CAPS2	Calcyphosin-2	63795.55	5.960457	0	0	0	1	0	0	0	0
MRPL13	39S rib omal protein L13, mitochondrial	20678.68	9.18	1	0	0	0	0	0	0	0
NLN	Neurolysin, mitochondrial	80599.69	4.829857	0	0	0	0	0	1	0	0
PRSS22	Brain-specific serine protease 4	33710.1	6.12249	0	0	0	0	0	1	0	0
WNT10A	Protein Wnt-10a	46414.11	9.38	1	0	0	0	0	0	0	0
C11orf54	Ester hydrolase C11orf54	35094.61	5.189705	0	0	0	0	0	1	0	0
	SDC1	Syndecan -1	32456.83	0	1	0	0	0	0	0	0
SH3BGRL3	SH3 domain-binding glutamic acid-rich-like protein 3	10431.26	4.82	0	0	0	0	1	0	0	0
ACBD3	Golgi resident protein GCP60	60555.98	5.02	0	0	0	0	1	0	0	0
PTPN23	Tyr ine-protein ph phatase non-receptor type 23	178861	6.45	1	0	0	0	0	0	0	0
EPB41L1	Band 4.1-like protein 1	98442.17	4.931969	0	0	0	0	0	0	1	0
PPP1R3E	Protein ph phatase 1 regulatory subunit 3E	30624.96	11.16	0	0	0	0	1	0	0	0
EPB41L4A	Band 4.1-like protein 4A	69332.01	6.593956	0	0	0	0	0	0	1	0
ISYNA1	Inositol-3-phosphate synthase 1	61029.1	4.83947	0	0	0	0	0	0	0	1
SPINK5	Serine protease inhibitor Kazal-type 5	120636.6	6.836525	0	0	1	0	0	0	0	0
NIT2	Nitrilase homolog 2	30588.56	4.541786	0	0	0	0	0	1	0	0
AASDHPPT	L-aminoadipate-semialdehyde dehydrogenase-phosphopantetheinyl transferase	35753.07	5.651344	0	0	0	0	0	1	0	0
HEBP1	Heme-binding protein 1	21083.52	4.518608	0	0	0	0	0	1	0	0
KRT82	Keratin, type II cuticular Hb2	56616.32	5.039848	0	0	0	1	0	0	0	0

MAN2C1	Alpha-mannosidase 2C1	115761.5	5.343941	0	0	0	0	0	1	0	0
SEPT11	Septin-11	49367.19	6.36	0	0	0	0	1	0	0	0
POLR3E	DNA-directed RNA polymerase III subunit RPC5	79847.41	5.096779	0	0	0	0	0	1	0	0
ADPRHL2	Poly(ADP-rib e) glycohydrolase ARH3	38922.17	4.95	0	0	0	0	1	0	0	0
LRP1B	Low-density lipoprotein receptor-related protein 1B	515159.4	5.09	1	0	0	0	0	0	0	0
RRBP1	Ribosome-binding protein 1	152380.7	8.126701	0	0	0	0	0	0	1	0
SAE1	SUMO-activating enzyme subunit 1	38425.56	4.847374	0	0	0	0	0	0	0	1
MRC2	C-type mannose receptor 2	166548.9	4.793221	0	0	1	0	0	0	0	0
CPNE7	Copine-7	70249.18	5.97	1	0	0	0	0	0	0	0
VPS29	Vacuolar protein sorting-associated protein 29	20492.68	5.033974	0	0	1	0	0	0	0	0
GRHPR	Glyoxylate reductase/hydroxypyruvate reductase	35645.74	5.925102	0	0	0	1	0	0	0	0
FBLN5	Fibulin-5	50146.67	3.601524	0	0	0	0	0	0	0	1
KLK11	Kallikrein-11	31038.78	8.915184	0	0	1	0	0	0	0	0
SRP68	Signal recognition particle 68 kDa protein	70685.56	5.571236	0	0	1	0	0	0	0	0
STEAP1	Metalloreductase STEAP1	39825.43	6.296486	0	0	0	0	0	0	0	1
PUF60	Poly(U)-binding-splicing factor PUF60	59837.62	4.733727	0	0	0	0	0	1	0	0
C21orf104	Putative uncharacterized C21orf104 protein	16526.34	5.39222	0	0	0	0	0	0	0	1
	MARCKS	Myristoylated alanine-rich C-kinase substrate	31535.86	0	1	0	0	0	0	0	0
TMPRSS11E	Transmembrane protease, serine 11E	47665.08	8.85	1	0	0	0	0	0	0	0
DNPEP	Aspartyl aminopeptidase	52394.92	6.175362	0	0	0	0	0	1	0	0
SLC7A7	Y+L amino acid transporter 1	55954.25	3.889488	0	0	0	0	0	0	1	0

COL17A1	Collagen alpha-1(XVII) chain	150326.5	5.470726	0	0	1	0	0	0	0	0
PRPF19	Pre-mRNA-processing factor 19	55146.34	5.345436	0	0	0	0	0	1	0	0
NDRG2	Protein NDRG2	40772.18	5.08	0	0	0	0	1	0	0	0
RPH3AL	Rab effector Noc2	34442.33	8.886024	0	0	0	0	0	1	0	0
CLCA2	Calcium-activated chloride channel regulator 2	103875.5	5.218758	0	0	1	0	0	0	0	0
NCKAP1	Nck-associated protein 1	128706.5	6.18	1	0	0	0	0	0	0	0
GDA	Guanine deaminase	50970.57	4.694527	0	0	0	0	0	1	0	0
LSM2	U6 snRNA-associated Sm-like protein LSm2	10827.63	4.674446	0	0	0	0	0	0	0	1
CAB39	Calcium-binding protein 39	39843.74	6.43	0	0	0	0	1	0	0	0
F11R	Junctional adhesion molecule A	32562.49	4.812019	0	0	1	0	0	0	0	0
CLIC4	Chloride intracellular channel protein 4	28753.74	4.823128	0	0	0	0	0	1	0	0
AP1M2	AP-1 complex subunit mu-2	48078.17	8.23	0	0	0	0	1	0	0	0
FCGBP	IgGFc-binding protein	571638.8	4.395241	0	0	0	0	0	1	0	0

Table S5. The list of proteins previously reported in the serum of head and neck cancer patients

Protein name	Gene name	Swissprot AC	Specimen
Interleukin-8	IL-8	P10145	saliva, serum
Interleukin-6	IL-6	P05231	saliva, serum
Matrix metalloproteinase-2	MMP-2	P08253	serum
Matrix metalloproteinase-3	MMP-3	P08254	serum
Pro-epidermal growth factor	EGF	P01133	serum
Alpha-fetoprotein	AFP	P02771	serum
Keratin-19	KRT19	P08727	serum
Galectin-3-binding protein	M2BP	Q08380	saliva
Protein S100-A9	S100A9	P06702	saliva
CD59 glycoprotein	CD59	P13987	saliva
Catalase	CAT	P04040	saliva
Profilin-1	PFN1	P07737	saliva
Matrix metalloproteinase-13	MMP13	P45452	serum
Actin, alpha skeletal muscle	ACTA1	P68133	saliva
Myosin-1	MYH1	P12882	saliva
Epidermal growth factor receptor	EGFR	A8K2T7	serum
Signal transducer and activator of transcription 3	STAT3	P40763	saliva
Thioredoxin-dependent peroxide reductase, mitochondrial	PRDX3	P30048	saliva
Serpin B3	SERPINB3	P29508	saliva
Fibrinogen alpha chain	FGA	P02671	plasma
Ig gamma-3 chain C region	IGHG3	P01860	plasma
Ig Kappa chain C region	IGKC	P01834	plasma
Heat shock 70 kDa protein 1A/1B	HSPA1A	P08107	serum
SICAM-1	sICAM1	Q99930	serum
Serum amyloid A-1 protein	SAA1	P0DJI8	serum

Reference

[1] K. D. Lee, H . S. Lee, C .H. Jeon, Body fluid biomarkers for early detection of head and neck squamous cell carcinomas. Anticancer Res 31(2011), 1161-1167.

[2] T. B. Schaaij-Visser, R. H. Brakenhoff, C. R. Leemans, A. J. Heck, M. Slijper, Protein biomarker discovery for head and neck cancer. J Proteomics 73 (2010), 1790-1803.

[3] T. M. Rezende, M. de Souza Freire, O. L. Franco, Head and neck cancer: proteomic advances and biomarker achievements. Cancer 116 (2010), 4914-4925.

Table S6. Forty-nine candidate biomarkers of laryngeal SCC in the "secretory/releasing" database

Protein name	Gene name	Swissprot AC	Subcellular location
Serum albumin	ALB	P02768	Secreted
Gelsolin	GSN	P06396	Cytoplasm. Secreted
Protein disulfide-isomerase	P4HB	P07237	Endoplasmic reticulum lumen. Melanosome. Cell membrane
Annexin A2	ANXA2	P07355	Secreted. Melanosome
Thrombospondin-1	THBS1	P07996	Secreted
Vinculin	VCL	P18206	Cytoplasm. Cell junction. Cell membrane
Lumican	LUM	P51884	Secreted
Transforming growth factor-beta-induced protein ig-h3	TGFBI	Q15582	Secreted
Protein S100-A8	S100A8	P05109	Secreted. Cytoplasm. Cell membrane
Plasminogen activator inhibitor 1	SERPINE1	P05121	Secreted
Epithelial cadherin	CDH1	P12830	Cell junction. Cell membrane. Endosome. Golgi apparatus
Tenascin	TNC	P24821	Secreted
Calreticulin	CALR	P27797	Endoplasmic reticulum lumen. Cytoplasm. Secreted. Cell surface.
Metalloproteinase inhibitor 1	TIMP1	P01033	Secreted
Matrix metalloproteinase-9	MMP9	P14780	Secreted
Tenascin-X	TNXB	P22105	Secreted
von Willebrand factor	VWF	P04275	Secreted
Glutathione peroxidase 3	GPX3	P22352	Secreted
Chitinase-3-like protein 1	CHI3L1	P36222	Secreted
Transgelin-2	TAGLN2	P37802	nuclear membrane. Plasma membrane
Polymeric immunoglobulin receptor	PIGR	P01833	Cell membrane. Secreted
Interleukin-1 receptor antagonist protein	IL1RN	P18510	Secreted. Cytoplasm
Antithrombin-III	SERPINC1	P01008	Secreted
Alpha-1-antichymotrypsin	SERPINA3	P01011	Secreted
Vitronectin	VTN	P04004	Secreted

Plasma protease C1 inhibitor	SERPING1	P05155	Secreted
Collagen alpha-2(I) chain	COL1A2	P08123	Secreted
Leukocyte elastase	ELA2	P08246	Cell surface. Secreted
Monocyte differentiation antigen CD14	CD14	P08571	Cell membrane
SPARC	SPARC	P09486	Secreted
Thioredoxin	TXN	P10599	Nucleus. Cytoplasm. Secreted
Tryptase alpha-1	TPSAB1	P15157	Secreted
Metalloproteinase inhibitor 2	TIMP2	P16035	Secreted
Pigment epithelium-derived factor	SERPINF1	P36955	Secreted
Kallikrein-6	KLK6	Q92876	Secreted. Nucleus. Cytoplasm. Mitochondrion. Microsome
Insulin	INS	P01308	Secreted
Beta-2-glycoprotein 1	APOH	P02749	Secreted
Alpha-2-HS-glycoprotein	AHSG	P02765	Secreted
Alpha-1B-glycoprotein	A1BG	P04217	Secreted
Amyloid beta A4 protein	APP	P05067	Membrane
Trypsin-1	PRSS1	P07477	Secreted
Clusterin	CLU	P10909	Secreted
Versican core protein	VCAN	P13611	Secreted
Inter-alpha-trypsin inhibitor heavy chain H2	ITIH2	P19823	Secreted
Fibulin-1	FBLN1	P23142	Secreted
Zinc-alpha-2-glycoprotein	AZGP1	P25311	Secreted
Thrombospondin-2	THBS2	P35442	Secreted
Dermcidin	DCD	P81605	Secreted
Procollagen C-endopeptidase enhancer 1	PCOLCE	Q15113	Secreted

Table S7. The performance of KLK6 in distinguishing cancer cases from controls at selected cutoff points

Application	Cutoff (ng/ml)	Sensitivity (%)	Specificity (%)	Early stage* (%)
C-H	7.31	29.5	90.3	27.7
C-B	6.28	36.2	82.1	41.5
C-NC	6.34	35.6	82.2	40.0

*comprised of cases with stage I and stage II.

C-H: distinguish cancer cases from healthy cases; C-B: distinguish cancer cases from benign cases; C-NC: distinguish cancer cases from non-cancer cases (including healthy cases and benign cases).
