# Supplementary figures and images for: Identification of Human Tissue Kallikrein 6 as a Potential Marker of Laryngeal Cancer Based on the Relevant Secretory/Releasing Protein Database
Source: Dis Markers. 2014 Feb 11;2014:594093. doi: 10.1155/2014/594093 (PMC3942293; doi:10.1155/2014/594093)

0h

48h

A

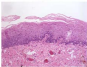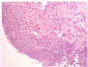

B

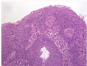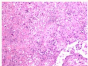



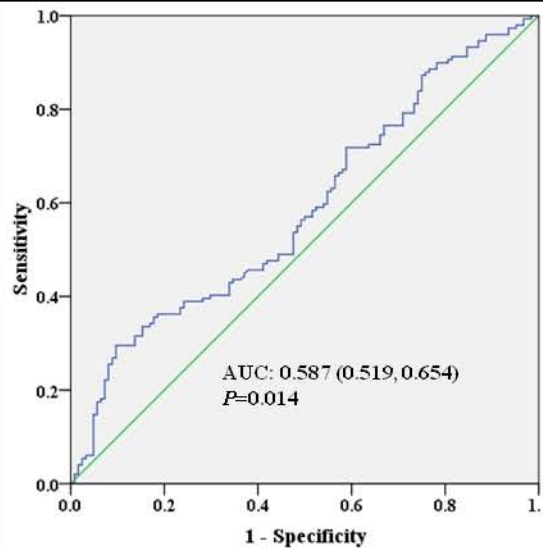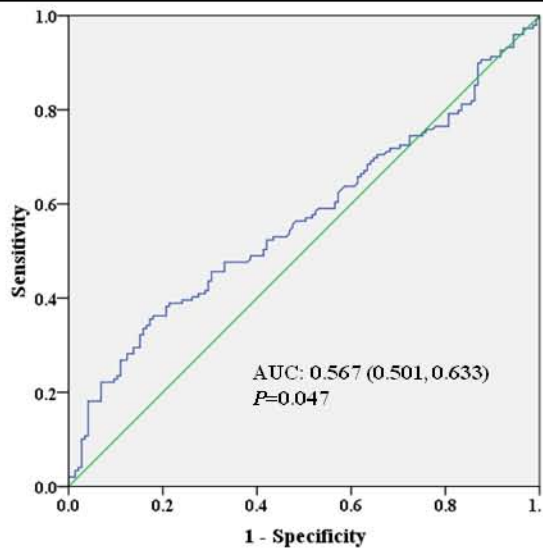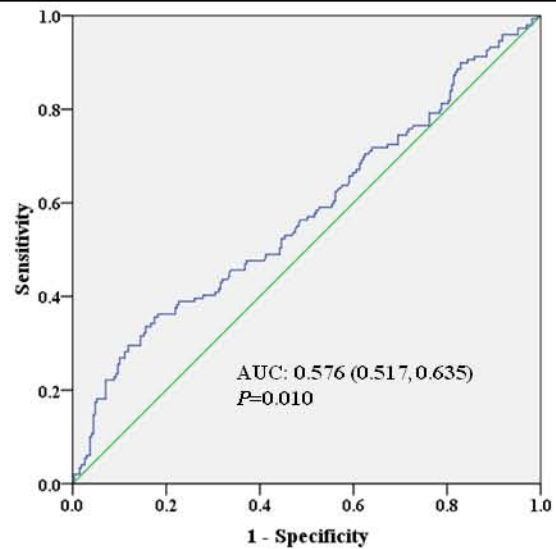

Supplement: Supplementary file 2 [file 594093.f2.pdf]
